# DDX17 helicase promotes resolution of R-loop-mediated transcription–replication conflicts in human cells

**DOI:** 10.1093/nar/gkac1116

**Published:** 2022-12-01

**Authors:** Barbora Boleslavska, Anna Oravetzova, Kaustubh Shukla, Zuzana Nascakova, Oluwakemi Ngozi Ibini, Zdenka Hasanova, Martin Andrs, Radhakrishnan Kanagaraj, Jana Dobrovolna, Pavel Janscak

**Affiliations:** Institute of Molecular Genetics of the Czech Academy of Sciences, Videnska 1083, 142 20 Prague 4, Czech Republic; Faculty of Science, Charles University in Prague, Albertov 6, 128 00 Prague 2, Czech Republic; Institute of Molecular Genetics of the Czech Academy of Sciences, Videnska 1083, 142 20 Prague 4, Czech Republic; Faculty of Science, Charles University in Prague, Albertov 6, 128 00 Prague 2, Czech Republic; Institute of Molecular Genetics of the Czech Academy of Sciences, Videnska 1083, 142 20 Prague 4, Czech Republic; Institute of Molecular Genetics of the Czech Academy of Sciences, Videnska 1083, 142 20 Prague 4, Czech Republic; School of Life Sciences, University of Bedfordshire, Park Square, Luton LU1 3JU, UK; Institute of Molecular Genetics of the Czech Academy of Sciences, Videnska 1083, 142 20 Prague 4, Czech Republic; Institute of Molecular Genetics of the Czech Academy of Sciences, Videnska 1083, 142 20 Prague 4, Czech Republic; School of Life Sciences, University of Bedfordshire, Park Square, Luton LU1 3JU, UK; School of Life Sciences, University of Westminster, 115 New Cavendish Street, London W1W 6UW, UK; Centre for Drug Discovery and Development, Sathyabama Institute of Science and Technology, Chennai 600119, India; Institute of Molecular Genetics of the Czech Academy of Sciences, Videnska 1083, 142 20 Prague 4, Czech Republic; Institute of Molecular Genetics of the Czech Academy of Sciences, Videnska 1083, 142 20 Prague 4, Czech Republic; Institute of Molecular Cancer Research, University of Zurich, Winterthurerstrasse 190, 8057 Zurich, Switzerland

## Abstract

R-loops are three-stranded nucleic acid structures composed of an RNA:DNA hybrid and displaced DNA strand. These structures can halt DNA replication when formed co-transcriptionally in the opposite orientation to replication fork progression. A recent study has shown that replication forks stalled by co-transcriptional R-loops can be restarted by a mechanism involving fork cleavage by MUS81 endonuclease, followed by ELL-dependent reactivation of transcription, and fork religation by the DNA ligase IV (LIG4)/XRCC4 complex. However, how R-loops are eliminated to allow the sequential restart of transcription and replication in this pathway remains elusive. Here, we identified the human DDX17 helicase as a factor that associates with R-loops and counteracts R-loop-mediated replication stress to preserve genome stability. We show that DDX17 unwinds R-loops *in vitro* and promotes MUS81-dependent restart of R-loop-stalled forks in human cells in a manner dependent on its helicase activity. Loss of DDX17 helicase induces accumulation of R-loops and the formation of R-loop-dependent anaphase bridges and micronuclei. These findings establish DDX17 as a component of the MUS81–LIG4–ELL pathway for resolution of R-loop-mediated transcription–replication conflicts, which may be involved in R-loop unwinding.

## INTRODUCTION

During genome duplication, the DNA replication machinery faces numerous obstacles, which can slow or stall replication fork progression (1). Failure to overcome these barriers to DNA replication can lead to genomic instability, a hallmark of cancer (1,2). Replication fork stalling can follow head-on transcription–replication encounters, which occur frequently after activation of oncogenes, leading to a phenomenon termed oncogene-induced replication stress (3). Studies in bacteria and human cells have shown that such transcription–replication conflicts (TRCs) promote the formation of co-transcriptional R-loops, which can impair replication fork progression (4–6). R-loops are formed by annealing of the nascent transcript to the template DNA strand behind the transcription complex, leaving the non-template DNA strand exposed as a single-stranded DNA (ssDNA) loop. This reaction is facilitated by the generation of underwound DNA in the wake of the elongating RNA polymerase complex and occurs favorably in the regions containing a high density of Gs in the non-transcribed strand (7,8). Evidence suggests that G-quadruplex (G4) structures formed in this strand stabilize the R-loop and promote its extension (9).

Our recent work has shown that replication fork stalling by co-transcriptional R-loops is an active process involving a fork remodeling reaction, termed replication fork reversal (6), which is mediated by RAD51 recombinase and the DNA translocases ZRANB3, SMARCAL1 and HLTF (10–13). Moreover, we have found that R-loop-induced fork reversal is antagonized by poly (ADP-ribose) polymerase (PARP)-regulated replication restart mediated by RECQ5 DNA helicase, MUS81-EME1 endonuclease, SLX4 scaffold protein, the DNA ligase IV(LIG4)–XRCC4 complex, the transcription elongation factor ELL, and active RNA synthesis. Detailed analysis of the underlying molecular mechanism suggested a model where fork cleavage-religation cycles catalyzed by MUS81–EME1 and LIG4–XRCC4 relieve the torsional stress in the DNA template generated by TRC, allowing ELL-dependent bypass of the transcription complex across the replication-stalling site and subsequent replication restart (6). However, how R-loops at TRC sites are eliminated to allow for the sequential restart of transcription and replication remains elusive.

Here, we employed mass spectrometry (MS)-based proteomics to identify the proteins that operate on the R-loop-forming transcription complexes at sites of TRCs and analyze their role in TRC resolution. Our experiments highlight DDX17 helicase as a potential genome caretaker during transcription-induced replication stress. Our findings suggest that DDX17 might be involved in R-loop unwinding at sites of TRCs to promote replication restart *via* the MUS81–LIG4–ELL axis.

## MATERIALS AND METHODS

### Plasmid constructions

DDX17 cDNA (p72) cloned in pSG5-Myc vector (14) was used as a template for PCR amplifications of the DDX17 coding region. To construct a translation fusion between DDX17 and a self-cleaving affinity tag composed of a Mxe intein fragment and chitin biding domain (CBD) in the bacterial expression vector pTXB3 (NEB), the 5′- and 3′-portions of the DDX17 coding region overlapping in FspI site were amplified by PCR using the following pairs of primers: 5′-CGAATTCCATGGGCCGCGGAGGAGGCTTTGG-3′and 5′-ATAGCTGGCCAACCATCTC-3′, which amplify the 5′-portion and introduce NcoI and FspI sites, respectively; and 5′-AAGGCCGCTCTTCCGCACATTTTACGTGAAGGAGGAGGAGG-3′ and 5′-TGTGATGATCTGACTCGAAGG-3′, which amplify the 3′-portion and introduce FspI and SapI sites, respectively. After digestion with appropriate restriction enzymes, DDX17 fragments were inserted between the NcoI and SapI sites of pTXB3 vector, resulting in pTXB3-DDX17 construct. To clone the DDX17 coding region in pAIO vector in frame with a C-terminal Flag tag (15), it was amplified using the following primers: 5′-GTAGTACTTAAGATGCGCGGAGGAGGCTTTG-3′, 5′-GATATCGGATCCTTTACGTGAAGGAGGAGGAGG-3′, which introduce AflII and BamHI sites, respectively. After digestion with AflII and BamHI, digested DDX17 fragment was then inserted between AflII and BamHI in pAIO vector, resulting in pAIO-DDX17 construct. Site-directed mutagenesis of the DDX17 ATP binding site (K142R) was performed using a QuickChange II Site-Directed Mutagenesis Kit (Agilent Technologies) with the following primers: 5′-CAGACTGGCTCTGGGAGAACGTTGGCGTATCTC-3′, 5′-GAGATACGCCAACGTTCTCCCAGAGCCAGTCTG-3′.

### Cell culture

Human U-2 OS osteosarcoma cells (ATCC HTB-96), HeLa Kyoto cells (ATCC CVCL_1922), primary MRC-5 fibroblasts (ATCC CCL-171), hTERT-immortalized retinal pigment epithelial cells RPE-1 (ATCC CRL-4000), and U-2 OS T-REx (Invitrogen) cell lines carrying pAIO-based vectors were cultivated in Dulbecco's modified Eagle's medium (DMEM, Gibco) supplemented with 10% (v/v) fetal bovine serum (FBS, Tet-free approved, Gibco), 100 U/ml penicillin and 100 μg/ml streptomycin. Cells stably transfected with pAIO plasmids were selected in the presence of hygromycin B (50 μg/ml; Sigma-Aldrich, H3274) and puromycin [Sigma-Aldrich, P8833; 1 μg/ml for RNH1(WT/D210N)-GFP, 1 μg/ml for DDX17(WT/K142R)-FLAG, and 0.4 μg/mL for RNH1(D210N)-BioID2-HA]. Doxycycline (Takara Bio, 631311) was added for 24 h to induce the expression of RNH1(WT/D210N)-GFP (1 ng/ml) and DDX17(WT/K142R)-FLAG (0.5 ng/ml) and for 12 h to induce the expression of RNH1(D210N)-BioID2-HA (0.2 ng/ml). All cells were grown at 37°C in humidified atmosphere in 5% CO_2_. For MS experiments, cells were treated with CPT (100 nM; Sigma-Aldrich, C9911) followed by treatment with biotin (50 μM; Sigma-Aldrich, B4501). For cell synchronization at the G2/M phase border, cells were treated with the CDK1 inhibitor RO-3306 (9 μM; Sigma-Aldrich, SML0569). For cytokinesis inhibition, cells were treated with cytochalasin B (2 μg/ml; Merck, C6762).

### Small-interfering RNA transfections

Transfections of siRNAs (a final concentration of 20–40 nM) were done using Lipofectamine RNAiMAX (Thermo Fisher, 13778150) according to the instructions of manufacturer. 24 h after siRNA transfection, the medium was exchanged with fresh medium. Experiments were typically carried out 72 h after siRNA transfection. The majority of siRNA oligonucleotides used in this study were purchased from Microsynth AG. The sequences of the sense strand of siRNA duplexes are: siLuc (CGUACGCGGAAUACUUCGA), siDDX17 (CCGGGAGCUACCAAUAUGAUA), siDDX17-2 (CGUAUCUCCUGCCUGCAAU), siDDX17-3 (CUGGAGUGCAUUUGAUAGUUA), siDHX9 (AAGGAGGAUGAUGGUGGUGAA), siDDX5 (ACAUAAAGCAAGUGAGCGAUU), siDHX15 (AGGGACUAUUAUAUUAAUAUA), siDDX3X (GAGGAACAUAAAUAUUACUAA), siMUS81 (CAGCCCUGGUGGAUCGAUA). ZRANB3 siRNA was purchased from Dharmacon (84083, D-010025-03-0005).

### Antibodies

The following antibodies were used for western blotting (WB), immunofluorescence staining (IF) or slot blot: anti-biotin (ab53494, Abcam; WB, 1:500), anti-RNase H1 (in-house made, rabbit polyclonal; WB, 1:2500), anti-TFIIH (sc-293, Santa Cruz Biotechnology; WB, 1:1000), anti-HA (MMS-101R, BioLegend; IF, 1:400), anti-DDX17 (sc-398168, Santa Cruz Biotechnology; WB, 1:500), anti-DHX9 (sc-66997, Santa Cruz Biotechnology; WB, 1:500), anti-DDX5 (sc-365164, Santa Cruz Biotechnology; WB, 1:200), anti-DHX15 (NB100-586, Bio-Techne; WB, 1:500), anti-DDX3X (sc-81247, Santa Cruz Biotechnology; WB, 1:500), anti-PCNA (ab18197, Abcam; IF, 1:200), anti-MUS81 (M1445, Sigma; WB, 1:2000), anti-ZRANB3 (23111–1-AP, Proteintech; WB, 1:1000), anti-tubulin (sc-53030, Santa Cruz Biotechnology; WB, 1:1000), anti-FLAG (F1804, Sigma; WB, 1:1000 and IF 1:200), anti-RNA:DNA hybrid antibody S9.6 (ENH001, Kerafast; slot blot, 1:1000), anti-ssDNA antibody (MAB3868, Millipore; slot blot, 1:10 000), anti-BrdU reacting with CldU (ab6326, Abcam; IF, 1:500), anti-BrdU reacting with IdU (347580, BD Biosciences; IF, 1:100), Cy3-conjugated donkey anti-rat secondary antibody (712-166-153, Jackson ImmunoResearch; IF, 1:150); Alexa Fluor (AF)-conjugated secondary antibodies (Life Technologies; IF, 1:400): AF 488 goat anti-mouse (A11001), AF 568 goat anti-mouse (a11031), AF 568 goat anti-rabbit (A11036), AF 647 goat anti-mouse (A21235), AF 647 goat anti-rabbit (a21245); horseradish peroxidase-coupled (HRP) secondary antibodies (WB, 1:5000): goat anti-rabbit (A0545, Sigma-Aldrich), goat anti-mouse (A4416, Sigma Aldrich).

### Western blotting

Harvested cells were resuspended in lysis buffer [50 mM Tris–HCl (pH 7.5), 120 mM NaCl, 0.5% (v/v) Nonidet P-40] supplemented with protease inhibitor cocktail (Roche, 11873580001), and incubated for 5 min on ice. Samples were sonicated, centrifuged (16 100 × g, 10 min, 4°C) and protein concentration was determined using the Bradford assay (Bio-Rad) or Bicinchoninic acid assay (Sigma, BCA1-1KT). 20–50 μg of total protein from the cell lysates were loaded onto 8–12% SDS-PAGE gel. After electrophoretic separation, proteins were transferred onto a nitrocellulose membrane (VWR international, 10600003) in a wet-transfer apparatus (Bio-Rad; 350 mA, 90 min, 4°C) with buffer containing 2.5 mM Tris–HCl, 10% (v/v) methanol and 19.2 mM glycine. After transfer, membranes were blocked with 3% milk in TBS-T [20 mM Tris–HCl (pH 7.5), 150 mM NaCl, 0.1% (v/v) Tween-20] for 30 min at RT and incubated with primary antibodies (diluted in 3% milk in TBS-T) overnight at 4°C. Membranes were then washed with TBS-T and incubated with appropriate horseradish peroxidase-conjugated secondary antibody (diluted in TBS-T) for 30 min at RT, and again washed with TBS-T. Protein bands were detected with the chemiluminescence reagent (Pierce ECL Western Blotting Substrate, Thermo Fisher, 32209). For detection of biotinylated proteins, the membranes were incubated with streptavidin labeled with an infrared dye (IRDye, 800CW Streptavidin; 926-32230; LI-COR; dilution 1:10000) and scanned using Odyssey 9120.

### Isolation of biotinylated proteins

To isolate biotinylated proteins from cell nuclei, trypsin-harvested cells (∼5 × 10^6^ per sample) were resuspended in 500 μl of fractionation buffer [10 mM HEPES–NaOH (pH 7.9), 10 mM KCl, 1.5 mM MgCl_2_, 0.34 M sucrose, 10% (v/v) glycerol, 1 mM DTT] supplemented with 1 mg/ml digitonin (Sigma-Aldrich, D141-500MG). After incubation on ice for 5 min, nuclei were collected by centrifugation (1500 × g, 10 min, 4°C), washed with fractionation buffer without digitonin and resuspended in 500 μl of lysis buffer [50 mM Tris–HCl (pH 7.5) 120 mM NaCl, 0.5% (v/v) Nonidet P-40] supplemented with protease inhibitor cocktail (Roche, 11873580001), and incubated for 5 min on ice. After sonication and subsequent centrifugation (16 100 × g, 10 min, 4°C), the supernatant was incubated for 30 min at room temperature (RT) with 10 μl of Dynabeads MyOne Streptavidin C1 (Invitrogen, 65002). Beads were washed with (i) 2% (w/v) sodium dodecyl sulphate (SDS); (ii) 0.1% (w/v) sodium deoxycholate, 1% (v/v) Triton X-100, 1 mM EDTA, 500 mM NaCl, 50 mM HEPES–NaOH (pH 7.5); (iii) 0.5% (w/w) sodium deoxycholate, 0.5% (v/v) Nonidet P-40, 1 mM EDTA, 250 mM LiCl, 10 mM Tris–Cl (pH 7.5); (iv) 50 mM Tris–Cl (pH 7.5), 120 mM NaCl. 75% of the beads from each sample were snap-frozen in liquid nitrogen and submitted to mass spectrometry analysis. 25% of the beads were boiled in 2× Laemmli sample buffer (95°C, 5 min) and the eluted proteins were subjected to western blot analysis.

### Mass spectrometry

Protein digestion and MS analyses were performed in the Laboratory of Mass Spectrometry [Biotechnology and Biomedicine Centre of the Academy of Sciences and Charles University (Biocev), Vestec, Czech Republic] as follows. Pull-down samples were resuspended in 100 mM triethylammonium bicarbonate containing 2% (w/v) sodium deoxycholate. Cystines were reduced with 5 mM final concentration of tris(2-chloroethyl) phosphate (60°C for 60 min) and blocked with 10 mM final concentration of *S*-methyl-methanethiosulfonate (10 min, RT). Samples were cleaved on beads (final volume 100 μl) with 1 μg of trypsin at 37°C overnight. After digestion, samples were centrifuged and supernatants were collected and acidified with trifluoroacetic acid to a final concentration of 1% (w/v). Sodium deoxycholate was removed by extraction with ethylacetate (16). Peptides were desalted using in-house made stage tips packed with C18 disks (Empore) according to Rappsilber et al. (17).

Nano Reversed phase column (EASY-Spray column, 50 cm × 75 μm ID, PepMap C18, 2 μm particles, 100 Å pore size) was used for LC/MS analysis. Mobile phase buffer A was composed of water and 0.1% (v/v) formic acid. Mobile phase B was composed of acetonitrile and 0.1% (v/v) formic acid. Samples were loaded onto the trap column (Acclaim PepMap300 C18, 5 μm, 300 Å Wide Pore, 300 μm × 5 mm) for 4 min at 15 μl/min. Loading buffer was composed of water, 2% (v/v) acetonitrile and 0.1% trifluoroacetic acid. Peptides were eluted with a Mobile phase B gradient from 4% to 35% B in 60 min. Eluting peptide cations were converted to gas-phase ions by electrospray ionization and analyzed on a Thermo Orbitrap Fusion (Q-OT- qIT, Thermo). Survey scans of peptide precursors from 350 to 1400 m/z were performed at 120k resolution (at 200 *m*/*z*) with a 5 × 10^5^ ion count target. Tandem MS was performed by isolation at 1.5 Th with the quadrupole, higher energy collisional dissociation (HCD) fragmentation with normalized collision energy of 30, and rapid scan MS analysis in the ion trap. The MS2 ion count target was set to 10^4^ and the max injection time was 35 ms. Only those precursors with charge state 2–6 were sampled for MS2. The dynamic exclusion duration was set to 45 s with a 10 ppm tolerance around the selected precursor and its isotopes. Monoisotopic precursor selection was turned on. The instrument was run in top speed mode with 2 s cycles (18).

All data were analyzed and quantified with the MaxQuant software (version 1.6.1.0) (19). The false discovery rate (FDR) was set to 0.1 for both proteins and peptides and we specified a minimum length of seven amino acids. The Andromeda search engine was used for the MS/MS spectra search against the *Human* database (downloaded from Uniprot on September 2017, containing 20 142 entries). Enzyme specificity was set as C-terminal to Arg and Lys, also allowing cleavage at proline bonds and a maximum of two missed cleavages. Dithiomethylation of cysteine was selected as fixed modification and N-terminal protein acetylation and methionine oxidation as variable modifications. The ‘match between runs’ feature of MaxQuant was used to transfer identifications to other LC–MS/MS runs based on their masses and retention time (maximum deviation 0.7 min) and this was also used in quantification experiments. Quantifications were performed with the label-free algorithms as described (19). Data analysis was performed using Perseus 1.6.2.3 software (20).

### DNA fiber spreading assay

Cells were labeled with 30 μM 5-chloro-2'-deoxyuridine (CldU) (C6891, Sigma-Aldrich) for 30 min, washed three times with PBS, and then labeled with 250 μM 5-iodo-2'-deoxyuridine (IdU) (I7125, Sigma-Aldrich) for 30 min. During the second labeling, cells were treated with 100 nM CPT. Where specified, cells were treated with 10 μM olaparib (S1060, Selleckchem), which was added 2 h prior CldU/IdU labeling and was also present during the labeling. Labeled cells were harvested and re-suspended in 1 × PBS to a concentration of 250 000 cells per ml and diluted 1:2 with unlabeled cells. 2.5 μl of this cell suspension were mixed with 7.5 μl of lysis buffer [200 mM Tris–HCl (pH 7.5), 50 mM EDTA, 0.5% (w/v) SDS] directly on a slide. After 9 min, slides were tilted at 30° causing the drops to run down by gravity. The DNA spreads were air-dried, fixed in methanol/acetic acid (3:1) at RT for 20 min. DNA was denatured with 2.5 M HCl for 1 h at RT, and slides were then washed four times with 1 × PBS and blocked with 2% (w/v) BSA in 1 × PBS/0.1% (v/v) Tween-20 (PBST) for 15 min. Slides were stained with primary antibodies (rat anti-CldU, mouse anti-IdU) for 2.5 h, washed with PBST, incubated with secondary antibodies (anti-rat Cy3, anti-mouse AF 488) for 2 h and washed with PBST. Slides were air-dried and mounted with Fluoromount-G mounting medium (00–4958-02, Thermo Scientific). DNA fiber images were acquired on Leica DM6000 upright fluorescent microscope (63×/1.40 oil immersion). CldU and IdU tract lengths were measured using ImageJ. Replication fork speed (kb/min) was calculated based on the assumption that 1 μm of DNA fiber corresponds to 2.59 kb (21).

### Immunofluorescence assays

Cells grown on autoclaved coverslips were fixed with 4% (v/v) paraformaldehyde in PBS for 15 min in the dark at RT, followed by permeabilization with 0.1% (v/v) Triton X-100 for 10 min at RT. For visualization of RNH1(D210N)-GFP foci, cells were permeabilized with pre-extraction buffer [25 mM HEPES–NaOH (pH 7.7), 50 mM NaCl, 1 mM EDTA, 3 mM MgCl_2_, 0.3 M sucrose, 0.5% (v/v) Triton X-100] for 5 min on ice, followed by fixation with 4% (v/v) paraformaldehyde. For PCNA staining, fixation with 4% (v/v) paraformaldehyde was followed by fixation with ice-cold methanol for 20 min at –20°C. After washing with 1× PBS, coverslips were blocked with 1% (w/v) BSA in PBS for 10 min at RT, stained with primary antibodies for 90 min, subsequently washed with 1 × PBS, and incubated with appropriate Alexa Fluor-conjugated secondary antibodies. For staining biotinylated proteins, streptavidin-phycoerythrin conjugate (12-4317-87, eBioscience; dilution 1:200) was used. Finally, cell nuclei were counterstained with 406-diamidine-20-phenylindole (DAPI) (D9542-10MG, Sigma-Aldrich; 1 μg/ml) and mounted using Fluoromount-G mounting media. Cell images were acquired on Leica DM6000 upright fluorescent microscope (63×/1.40 oil immersion) and on an IX81 microscope (Olympus) equipped with ScanR imaging platform using a 60×/1.4 NA objective with oil immersion. Images were analyzed with ScanR Analysis software based on PCNA/FLAG/HA signal intensity or using spot detector plugin to detect RNH1(D210N)-GFP foci. Approximately 1700 cells per condition were measured. Nuclei were identified based on the DAPI signal. To prepare the scatter plot in Figure 3D, the values obtained were exported to the Tibco Spotfire software (Tibco Spotfire 11.8.0).

### Detection of RNA:DNA hybrids with S9.6 antibody

For detection of RNA:DNA hybrids by slot blot, harvested cells were resuspended in lysis buffer [40 mM Tris–HCl (pH 7.5), 1.28 M sucrose, 20 mM MgCl_2_, 4% (v/v) Triton X-100] and centrifuged (1300 × g, 4°C, 15 min). Isolated nuclei were incubated in digestion buffer [30 mM Tris–HCl (pH 8.0), 800 mM guanidine, 30 mM EDTA, 5% (v/v) Tween 20, 0.5% (v/v) Triton X-100] supplemented with proteinase K (0.74 mg/ml) at 50°C for 2 h. Nucleic acids were extracted using chloroform, precipitated with ethanol and dissolved in TE buffer [10 mM Tris–HCl (pH 8), 1 mM EDTA]. Indicated samples (3 μg of DNA) were incubated with 5 U of RNase H (NEB, M0297) at 37°C for 3 h. The DNA samples (0.5 and 1 μg) were spotted in duplicates onto nylon membranes (Hybond-N^+^, RPN203B, GE Healthcare) using a slot blot manifold (CSL-S48, Cleaver Scientific). For total DNA, the membrane was denatured in a solution containing 0.5 M NaOH and 1.5 M NaCl for 10 min and subsequently neutralized in 0.5 M Tris–HCl (pH 7.2) containing 1.5 M NaCl for 10 min. The membranes were UV-crosslinked (0.12 J/m^2^) and immunostained with S9.6 antibody (1:1000) or mouse monoclonal anti-ssDNA antibody (1:10 000, MAB3868 Millipore), and goat anti-mouse IgG-HRP (Sigma-Aldrich, A4416) to detect RNA:DNA hybrids and total DNA, respectively, with chemiluminescence reagent (Pierce ECL Western Blotting Substrate, Thermo Fisher, 32209). Signal quantification was performed using ImageJ Fiji64.

### Chromatin immunoprecipitation with S9.6 antibody

Cells grown on tissue culture plates were permeabilized with pre-extraction buffer [25 mM HEPES–NaOH (pH 7.7), 50 mM NaCl, 1 mM EDTA, 3 mM MgCl_2_, 0.3 M sucrose, 0.5% (v/v) Triton X-100] supplemented with protease inhibitor cocktail (Roche, 11873580001) and phosphatase inhibitor cocktail (Roche, 4906837001) for 5 min on ice and subsequently washed with pre-extraction buffer without Triton X-100. After washing the plates with PBS, cells were scraped into lysis buffer [50 mM Tris–HCl (pH 8.0), 100 mM NaCl, 1% (v/v) NP-40] supplemented with protease and phosphatase inhibitor cocktail and incubated 10 min on ice. Chromatin DNA was sheared to 800–200 bp in size by sonication 16 × 30 s ON/30 s OFF in 4°C (Covaris), followed by centrifugation (16 100 × g, 4°C, 10 min). Where indicated, supernatants were supplemented with 3 mM MgCl_2_, and treated with 5 μl of RNaseH (NEB, M0297L) for 45 min at 37°C. Samples were then incubated with 2 μg of S9.6 antibody or control IgG overnight at 4°C. After incubation, samples were supplemented with 20 μl of protein A/G magnetic beads (ThermoFisher Scientific, 26162) and incubated for 2 h at 4°C. Beads were washed three times with wash buffer [20 mM Tris–HCl (pH 8.0), 100 mM NaCl, 0.5% Triton, 2 mM EDTA] supplemented with protease inhibitor cocktail, and boiled in 2× Laemmli sample buffer (95°C, 10 min). The eluted proteins were subjected to western blot analysis.

### DNA–RNA immunoprecipitation and quantitative real-time PCR

DNA–RNA immunoprecipitation (DRIP) experiments were carried out as described (22) with a few minor modifications for U-2 OS cells. Briefly, nucleic acids isolated from cells using conventional phenol-chloroform extraction were digested with restriction enzymes including BamHI, BsrGI, EcoRI, HindIII and XhoI (20 U each; Thermo Scientific) for 36 h at 37°C, either with or without the addition of RNaseH (40 U, NEB). Digested nucleic acids were cleaned by phenol-chloroform extraction and then resuspended in nuclease-free water (Sigma-Aldrich). Using 6.5 μg of S9.6 antibody in binding buffer [10 mM NaPO4 (pH 7.0), 140 mM NaCl and 0.05% (v/v) Triton X-100], RNA:DNA hybrids were immunoprecipitated from 2.5 μg of total nucleic acids overnight at 4°C. Immune complexes were subsequently incubated with Pierce™ protein A/G magnetic beads (Thermo Scientific, 88803) for 2 h at 4°C, washed two times in binding buffer and one time in TE buffer [10 mM Tris–HCl (pH 8.1), 1 mM EDTA] for 15 min at room temperature, and eluted with elution buffer [50 mM Tris–HCl (pH 8.0), 10 mM EDTA, 0.5% (w/v) SDS]. Proteinase K digestion was then performed for 30 min at 55°C, followed by phenol-chloroform extraction and the DRIP-derived nucleic acids were suspended in nuclease-free water. DRIP-derived samples were subjected to quantitative real-time PCR (qPCR) analysis using iQ SYBR Green Supermix reagent (Bio-Rad) and the gene-specific primers described below on a CFX96 Real-Time PCR detection system (Bio-Rad). All the data were analyzed using the 2^−ΔΔCT^ method (23). The percentage of DNA in the immunoprecipitates compared to the input DNA was calculated and plotted using GraphPad Prism software (version 9.3.1). The sequences of the primers used for qPCR are: 5′-CCGGTGAGAAGCGCAGTCGG-3′ (APOE-Forward); 5′-CCCAAGCCCGACCCCGAGTA-3′ (APOE-Reverse); 5′-GGCTGCTCAGGAGAGCTAGA-3′ (BTBD19-Forward); 5′-ACCAGACTGTGACCCCAAAG-3′ (BTBD19-Reverse); 5′-GCCTGTGAGTGAGTGCAGAA-3′ (GADD45A-Forward); 5′-CGACTCACCTTTCGGTCTTC-3′ (GADD45A-Reverse); 5′-AATGTGGCATTTCCTTCTCG-3′ (RPL13A-Forward); 5′-CCAATTCGGCCAAGACTCTA-3′ (RPL13A-Reverse); 5′-TGCCAGGAAGCCAAATGAGT-3′ (SNRPN-Forward); 5′-TCCCTCTTGGCAACATCCA-3′ (SNRPN-Reverse).

### Analysis of micronuclei

Cells grown on autoclaved coverslips were synchronized with cytochalasin B (2 μg/ml) for 16 h prior to harvest. Cells were fixed with 4% (v/v) paraformaldehyde in PBS for 15 min at RT, and then stained with DAPI (1 μg/ml). Cell images were acquired on an IX8 microscope (Olympus) equipped with ScanR imaging platform using a 60×/1.4 NA objective with oil immersion. The percentage of bi-nucleated cells with micronuclei was determined using ImageJ. At least 150 bi-nucleated cells were scored per condition in each experiment.

### Analysis of bulky anaphase bridges

Cells grown on autoclaved coverslips were synchronized with 9 μM RO-3306 for 16 h, followed by three washes with 1× PBS for 5 min at RT and subsequent incubation in DMEM medium for a total time of 1.5 h at 37°C. Cells were fixed with 4% (v/v) paraformaldehyde in PBS for 15 min at RT, followed by staining with DAPI (1 μg/ml). Cell images were acquired on an IX8 microscope (Olympus) equipped with ScanR imaging platform using a 60×/1.4 NA objective with oil immersion. The percentage of anaphase cells with bulky bridges was determined using ImageJ. At least 25 anaphase cells were scored per condition in each experiment.

### Protein purification

Wild-type and K142R forms of DDX17 were expressed in ArcticExpress RIL cells (Agilent Technologies) harboring pTXB3-DDX17 vectors. Cells were grown at 37°C in LB broth supplemented with ampicillin to reach an optical density of 0.4–0.5 at 600 nm. Protein expression was induced by addition of 1 mM isopropyl β-D-1-thiogalactopyranoside (IPTG) followed by incubation at 11°C for 24 h. Cells were harvested by centrifugation and resuspended in buffer A containing 50 mM Tris-Cl (pH 8), 500 mM NaCl, 1 mM EDTA, 10% (v/v) glycerol, 0,1% (v/v) Triton X-100 and 0.5 mM phenylmethanesulfonyl fluoride (PMSF). Cells were lysed by sonication at 40% amplitude in ice bath for 40 s in 10 cycles (Q125 ultrasonic processor, Qsonica). The lysate was clarified by centrifugation at 41 000 × g for 1 h at 4°C. The cleared supernatant was incubated with preequilibrated chitin resin (S6651S, Biotech) for 2 h at 4°C. Chitin resin was then washed 3 times with buffer A and once with buffer B [50 mM Tris–Cl (pH 8), 150 mM NaCl, 1 mM EDTA, 10% (v/v) glycerol]. Proteins were eluted with buffer B containing 80 mM DTT overnight at 4°C and were loaded on a 1 ml HiTrap heparin column (17-0406-01, VWR). Bound proteins were eluted in a 10-ml linear gradient of 150–1000 mM NaCl in buffer B supplemented with 1 mM DTT. The peak fractions were aliquoted, snap-frozen in liquid nitrogen and stored at -80°C. Concentration of purified proteins was estimated by measuring intensity of proteins bands on SDS-PAGE, using BSA as a standard.

### Preparation of RNA:DNA and DNA:DNA substrates

The synthetic oligonucleotides used for preparation of RNA:DNA and DNA:DNA substrates used in helicase assays were purchased from Microsynth AG (Switzerland). The sequences of these oligonucleotides and the schemes of the helicase substrates are shown in Supplementary Table S1. To prepare these substrates, unlabeled DNA oligonucleotides were mixed with 5’FAM-labeled RNA or DNA oligonucleotide at a molar ratio 1.5:1 in buffer containing 20 mM Tris (pH 7.5), 10 mM MgCl_2_, 2 mM DTT and 50 mM KCl (a total volume of 50 μl, the final concentration of labeled oligonucleotide was 100 nM). The mixtures were incubated at 95°C for 2 min and allowed to cool to RT overnight.

### Helicase assays

DNA/RNA substrates (5 nM) were incubated with DDX17 (in a 0–60 nM concentration range) in 10 μl of a helicase assay buffer containing 20 mM Tris–HCl (pH 7.5), 10 mM MgCl_2_, 2 mM DTT, 50 mM KCl and 5 mM ATP at 37°C for 20 min. ATP was added separately as the last component to start the reaction. Reactions were terminated by adding 3 μl of stop buffer containing 2% (w/v) SDS, 0.1% (w/v) bromophenol blue, xylene cyanol in 20% (v/v) glycerol, 0.1 mM EDTA, 5 mg/ml proteinase K and incubated 10 min at 37°C. The reaction products were separated by native PAGE (10–16%, 1× TBE). Gel images were acquired by Amersham Typhoon 5 (GE Healthcare) and quantified by ImageJ.

### ATPase assay

ATPase activity was determined by colorimetric estimation of the concentration of inorganic phosphate released by ATP hydrolysis using the malachite green assay in a 96-well microplate setup (24). 1 μM 45-mer DNA/RNA oligonucleotides (Supplementary Table S1) were incubated with 40 nM DDX17 in a buffer containing 20 mM Tris (pH 7.5), 10 mM MgCl_2_, 2 mM DTT and 50 mM KCl. Reactions were initiated by adding ATP to a final concentration of 2 mM and carried out for 60 min at 37°C. Reactions were terminated by addition of three reaction volumes of 0.1 M EDTA (pH 8.0).

### Statistical analysis

Statistical analysis was performed with GraphPad Prism 5.04 and 8.4.3 software. Mann–Whitney test and one-way ANOVA followed by Tukey's multiple comparisons test were used where appropriate. Statistical details of experiments can be found in the figure legends.

## RESULTS

### BioID-based proteomic analysis of R-loop interactome at sites of transcription–replication conflicts

**Figure 1.**
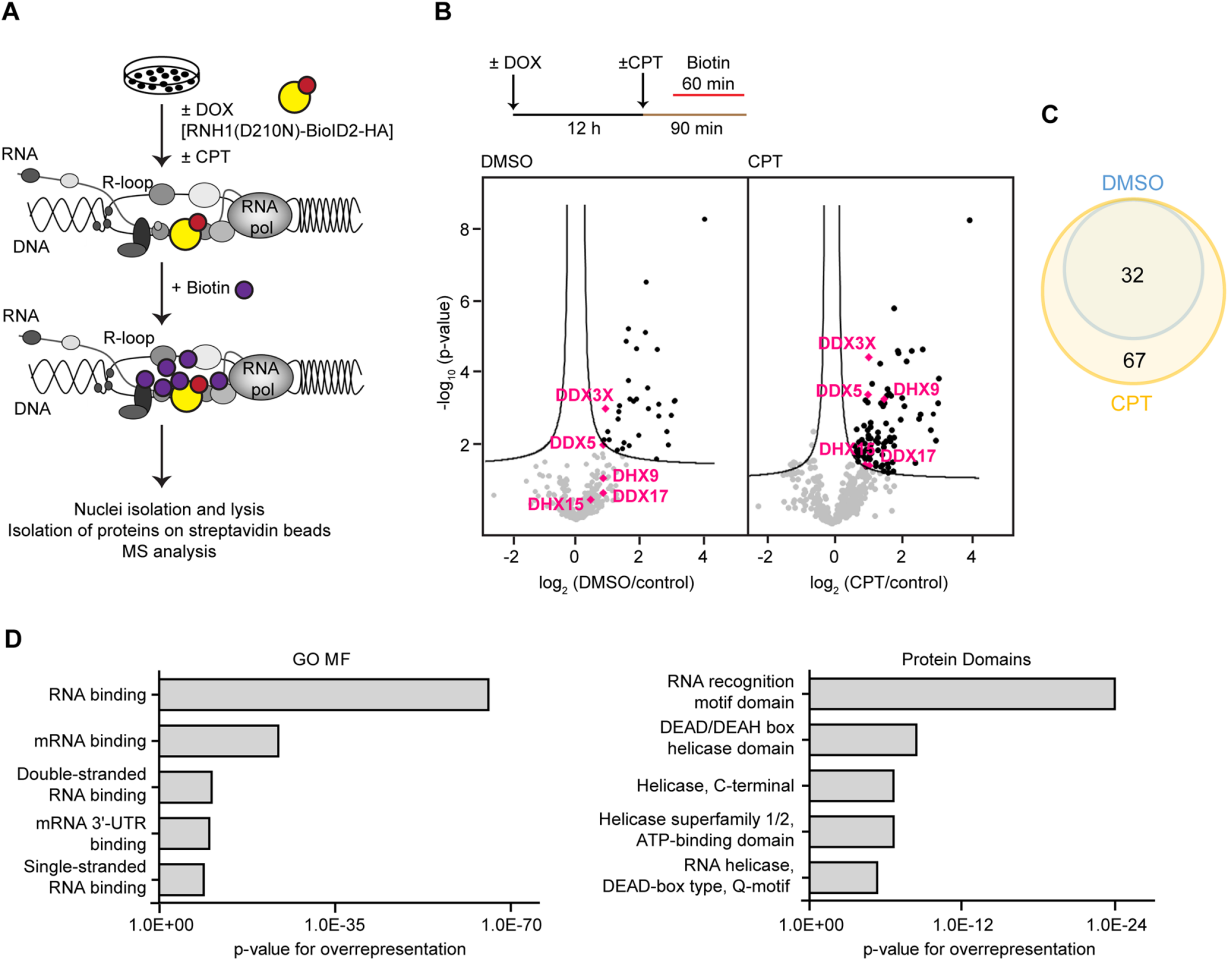
BioID-based proteomic analysis of R-loop interactome at sites of transcription–replication conflicts. (**A**) Schematic representation of BioID screen of proteins associated with R-loops. The expression of RNH1(D210N)-BioID2-HA fusion protein in U-2 OS T-REx RNH1(D210N)-BioID2-HA cells is induced by doxycycline. CPT (camptothecin, 100 nM) promotes the formation of co-transcriptional R-loops. (**B**) Top panel: Experimental workflow of protein biotinylation by RNH1(D210N)-BioID2-HA in living cells. Bottom panel: Volcano plots showing significantly differentially abundant proteins for comparison of data from RNH1(D210N)-BioID2-HA-expressing cells treated with vehicle alone (DMSO) or 100 nM CPT for 90 min to control cells in which expression of RNH1(D210N)-BioID2-HA was not induced (control). The -log_10_ (permutation-based FDR corrected P value) is plotted against the log_2_ (fold change: DMSO/control) or log_2_ (fold change: CPT/control). Each point represents the difference in abundance of particular protein between the two conditions plotted against the level of statistical significance. Data were processed using the Perseus program with false discovery rate (FDR) of 0.1 (black lines). Significantly enriched proteins are in the right upper quadrant of the volcano plot, depicted in black. DEAD/DEAH box helicases DDX17, DDX5, DHX15, DHX9 and DDX3X helicases are depicted in pink. (**C**) Venn diagram showing overlap of significantly enriched proteins in DMSO and CPT samples. (**D**) Significantly enriched proteins from BioID screen clustered into individual classes according to gene ontology molecular function (GO MF) annotations and protein domains. The x-axis indicates statistical significance of overrepresentation.

To identify the proteins, particularly helicases, that act on R-loops at TRC sites to promote replication restart, we utilized the BioID (25) method for proximity-dependent labeling of proteins in living cells (26). We made use of a previously established U-2 OS T-REx cell line conditionally expressing the promiscuous biotin ligase BioID2 fused to a catalytically-inactive form of RNase H1, which binds to but does not degrade RNA:DNA hybrids (27). In this chimeric protein, hereafter referred to as RNH1(D210N)-BioID2-HA, the mutant RNase H1 targets BioID2 to R-loops in the cell nuclei, which can then biotinylate the neighbouring proteins after the addition of biotin to the cell culture medium (Figure 1A; Supplementary Figure S1A–C). The biotinylated nuclear proteins can be isolated with streptavidin-coated beads and identified by mass spectrometry (MS) (Figure 1A). To increase the incidence of R-loop-mediated TRCs, we exposed cells to the DNA topoisomerase 1 inhibitor camptothecin (CPT), which promotes R-loop formation (6). By proximity ligation assay, we earlier demonstrated that exposure of cells to CPT increased colocalization between PCNA and RNA polymerase II (6), which indicates head-on TRCs (4). By quantitative image-based cytometry (QIBC) of pre-extracted cells, we observed that CPT treatment significantly increased the binding of RNH1(D210N)-BioID2-HA to chromatin (Supplementary Figure S1D), suggesting an elevated level of R-loops. For the proteomic analysis, the expression of RNH1(D210N)-BioID2-HA fusion protein was induced by addition of doxycycline at a concentration of 0.2 ng/ml and after 12 h, cells were treated with 100 nM CPT or vehicle alone (DMSO) for 90 min. Biotin (50 μM) was added into the cell culture medium for the last 60 min of the incubation time (Figure 1B). QIBC analysis showed that CPT treatment increased the level of biotinylation in cell nuclei (Supplementary Figure S1E). After the treatment, cells were harvested and fractionated to obtain intact nuclei, followed by nuclear lysis. Biotinylated proteins present in these extracts were captured by streptavidin-coated magnetic beads. The captured proteins were subjected to MS analysis for identification. As a control sample, we used cells in which the expression of RNH1(D210N)-BioID2-HA was not induced. Five biological replicates for induced and non-induced conditions were prepared and processed in parallel. The data were submitted to MaxQuant and analyzed using Perseus software, comparing mock-treated (DMSO), or CPT-treated (CPT) cells to the control condition. In total, 438 proteins were identified (Supplementary Table S2). Next, by using the moderate t-test, we identified 32 significantly enriched proteins for DMSO-treated cells and 99 proteins for CPT-treated cells when compared to the control (Figure 1B; Supplementary Table S3). Cross-comparison of the identified protein groups (DMSO vs. CPT) showed a complete overlap (Figure 1C). We also compared our results with the results of previous studies addressing the RNA:DNA hybrid interactome in unchallenged cells by: (i) chromatin immunoprecipitation using the S9.6 antibody (28,29); (ii) fractionation of cell extracts on affinity chromatography columns functionalized with RNA:DNA hybrids (30); (iii) R-loop-proximity proteomics using a fusion protein composed of the RNA:DNA hybrid-binding domain of RNase H1 and an engineered variant of ascorbate peroxidase (APEX2) (31). We found that a large part of the proteins in our dataset (from one half to one third) were also identified in the previous studies (Supplementary Figure S1F; Table S4). Finally, we clustered the identified proteins with significantly enriched abundance into individual classes according to gene ontology molecular function (GO MF) annotations and overexpressed protein domains (32). The biggest cluster of identified proteins was annotated as RNA-binding proteins (according to GO MF) and proteins with RNA recognition motif domain or DEAD/DEAH box helicase domain (according to protein domains) (Figure 1D).

### DDX17 promotes replication restart following R-loop-mediated fork stalling

**Figure 2.**
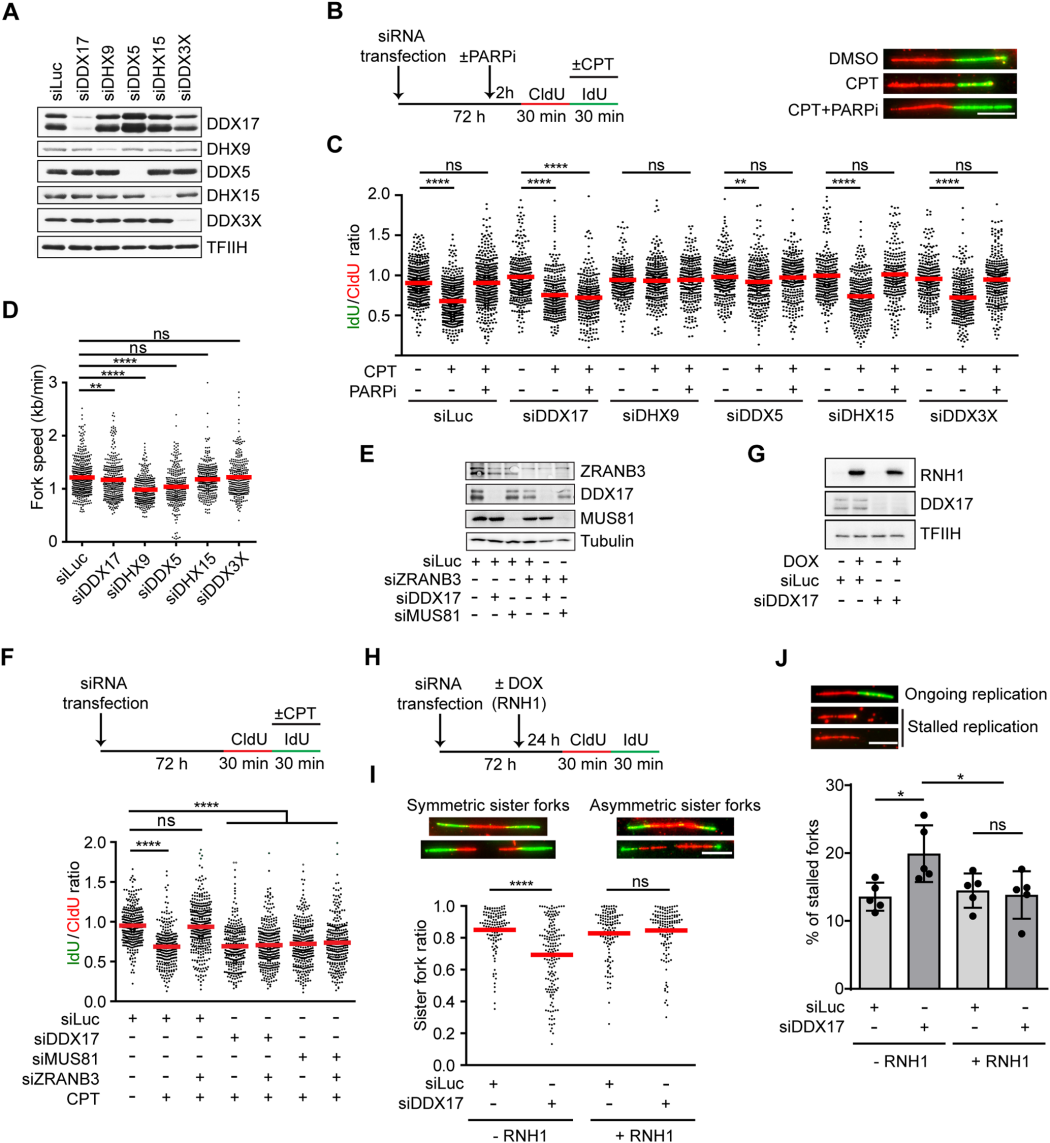
DDX17 promotes replication restart following R-loop-mediated fork stalling. (**A**) Western blot analysis of the lysates of U-2 OS cells transfected with indicated siRNAs. Note that DDX17 exists in two isoforms, p72 and p82, which result from alternative translation initiation sites. (**B**) Left panel: Experimental workflow of DNA fiber assays. U-2 OS cells were transfected with indicated siRNAs for 72 h followed by pulse-labeling with CldU and IdU. During the IdU labeling, cells were treated with 100 nM CPT or DMSO. Where indicated, 10 μM PARP inhibitor olaparib (PARPi) was added 2 h before labeling and was also present during the labeling. Right panel: Representative immunofluorescence images of replication tracts on DNA fibers of U-2 OS cells transfected with control siRNA (siLuc) and treated as indicated. Scale bar, 10 μm. (**C**) Effect of the depletion of DDX17, DHX9, DDX5, DHX15 and DDX3X respectively, on the rescue of CPT-induced replication fork slowing by PARP inhibition (PARPi). Scatter plot of the values of IdU/CldU tract length ratio obtained for indicated conditions (n ≥ 300) is shown. Red horizontal lines indicate mean value. ns, non-significant (*P* > 0.05); **P ≤ 0.01; ****P ≤ 0.0001 (Mann–Whitney test). (**D**) Effect of the depletion of DDX17, DHX9, DDX5, DHX15 and DDX3X, respectively, on replication fork velocity in U-2 OS cells (*n* ≥ 300). Red horizontal lines indicate mean value. ns, non-significant (*P* > 0.05); **P ≤ 0.01; ****P ≤ 0.0001 (Mann–Whitney test). (**E**) Western blot analysis of the lysates of U-2 OS cells transfected with indicated siRNAs. (**F**) DDX17 is required for the rescue of CPT-induced replication fork slowing by ZRANB3 depletion in U-2 OS cells. Top panel: Experimental workflow of DNA fiber assay. U-2 OS cells were transfected with indicated siRNAs for 72 h followed by pulse-labeling with CldU and IdU. During the second labeling, cells were treated with 100 nM CPT or DMSO. Bottom panel: Scatter plot of the values of IdU/CldU tract length ratio obtained for indicated conditions (*n* ≥ 300). Red horizontal lines indicate mean value. ns, non-significant (*P* > 0.05); ****P ≤ 0.0001 (Mann–Whitney test). (**G**) Western blot analysis of the lysates of U-2 OS T-REx [RNH1(WT)-GFP] cells transfected with indicated siRNAs. (**H**) Experimental workflow of DNA fiber assays with U-2 OS T-REx [RNH1(WT)-GFP] cells for (I) and (J). Expression of RNH1(WT)-GFP was induced by addition of doxycycline (DOX; 1 ng/ml) to eliminate R-loops. (**I**) Loss of DDX17 induces R-loop-dependent sister fork asymmetry. Top panel: representative images of symmetric and asymmetric replication tracts of sister forks. Scale bar, 10 μm. Bottom panel: scatter plot of the values of sister IdU tract length ratio (sister fork ratio; shorter IdU tract/longer IdU tract) measured for the indicated conditions (n ≥ 125). Red horizontal lines indicate mean value. ns, non-significant (*P* > 0.05); ****P ≤ 0.0001 (Mann–Whitney test). (**J**) Loss of DDX17 increases the frequency of stalled replication forks in an R-loop-dependent manner. Top panel: representative immunofluorescence images of replication tracts corresponding to ongoing (CldU tract is followed IdU tract) and stalled (CldU tract is not followed IdU tract) replication. Scale bar, 10 μm. Bottom panel: quantification of the replication fork stalling events. Data are mean ± SD, *n* = 5. ns, non-significant (*P* > 0.05); *P ≤ 0.05 (one-way ANOVA).

We next focused on the DEAD/DEAH box helicases found to be significantly enriched in the samples of CPT-treated cells, namely DDX17, DHX9, DDX5, DHX15 and DDX3X (Figure 1B). We investigated the impact of depletion of these helicases (Figure 2A–C) on the restart of replication forks blocked by CPT-induced R-loops in U-2 OS cells. Ongoing replication events were sequentially labeled with two halogenated thymidine analogues, namely 5-chloro 2′-deoxyuridine (CldU) and 5-iodo-2′-deoxyuridine (IdU), with CPT present during the second pulse-labeling with IdU (Figure 2B). The individual two-colour labeled replication tracts were visualized on DNA fiber spreads by immunofluorescence microscopy (Figure 2B). To boost replication restart *via* the MUS81–LIG4–ELL pathway, cells were pre-incubated for 2 h with the PARP inhibitor (PARPi) olaparib (10 μM), which counteracts R-loop-induced fork reversal by promoting RECQ1-mediated reverse branch migration (6,33). In agreement with previous reports, we found that PARPi treatment could completely rescue CPT-induced replication fork slowing in mock-depleted cells as revealed by measuring IdU/CldU tract length ratio (Figure 2B, C). Notably, out of the five helicases tested, we found that depletion of only DDX17 abrogated this unrestrained DNA synthesis, suggesting a role for this DEAD-box helicase in restarting R-loop-stalled forks (Figure 2B, C; Supplementary Figure S2A–C). In support of this notion, DDX17 was also found to be required for PARPi-mediated rescue of CPT-induced fork slowing in two non-cancerous human cell lines, namely MRC-5 and RPE-1 (Supplementary Figure S2D–G). On the contrary, depletion of DHX9 or DDX5 rather prevented the inhibitory effect of CPT on replication fork progression (Figure 2C), although their absence was associated with a marked reduction in replication fork velocity (Figure 2D). Of note, it has been shown that DHX9 promotes R-loop formation upon impairment of RNA splicing, possibly by unwinding secondary structures in the nascent transcripts (34). Similarly, we found that DHX9 was required for R-loop formation in CPT-treated cells (Supplementary Figure S3), explaining why its depletion rescues CPT-induced fork slowing (Figure 2C).

Based on the above analysis, we focused on DDX17 as a candidate helicase acting in the MUS81–LIG4–ELL pathway. To exclude the possibility that DDX17 promotes replication restart by counteracting fork reversal in conjunction with RECQ1, we evaluated its requirement for the rescue of CPT-induced fork slowing by depletion of the ZRANB3 DNA translocase, which mediates fork reversal (12). It has been shown that the unrestrained fork progression triggered in CPT-treated cells by abrogation of fork reversal activity depends upon MUS81, but not RECQ1 (6). We found that upon ZRANB3 depletion, CPT still induced replication fork slowing if cells were simultaneously depleted of DDX17 or MUS81 (Figure 2E, F).

To explore whether DDX17-depleted cells display R-loop-mediated replication stress without an external challenge, we compared the lengths of replication tracts of sister replication forks in U-2 OS T-REx cells which inducibly express wild-type RNase H1 (6). Cells were sequentially pulsed with CldU and IdU without or with induction of RNase H1 expression by doxycycline (Figure 2G, H). We observed that upon DDX17 depletion, the lengths of the IdU-labeled tracts of the sister replication forks displayed a marked asymmetry in cells grown in absence of doxycycline (Figure 2I), indicating replication fork stalling events. Consistently, DDX17-depleted cells also displayed increased incidence of CldU tracts without an IdU tract as compared to mock-depleted cells (Figure 2J), again indicating fork stalling events. Importantly, both these phenotypes could be completely rescued by RNase H1 overexpression (Figure 2G-J), suggesting that they are dependent on R-loop formation.

### Loss of DDX17 induces accumulation of R-loops during S-phase and the formation of R-loop-dependent anaphase bridges and micronuclei

**Figure 3.**
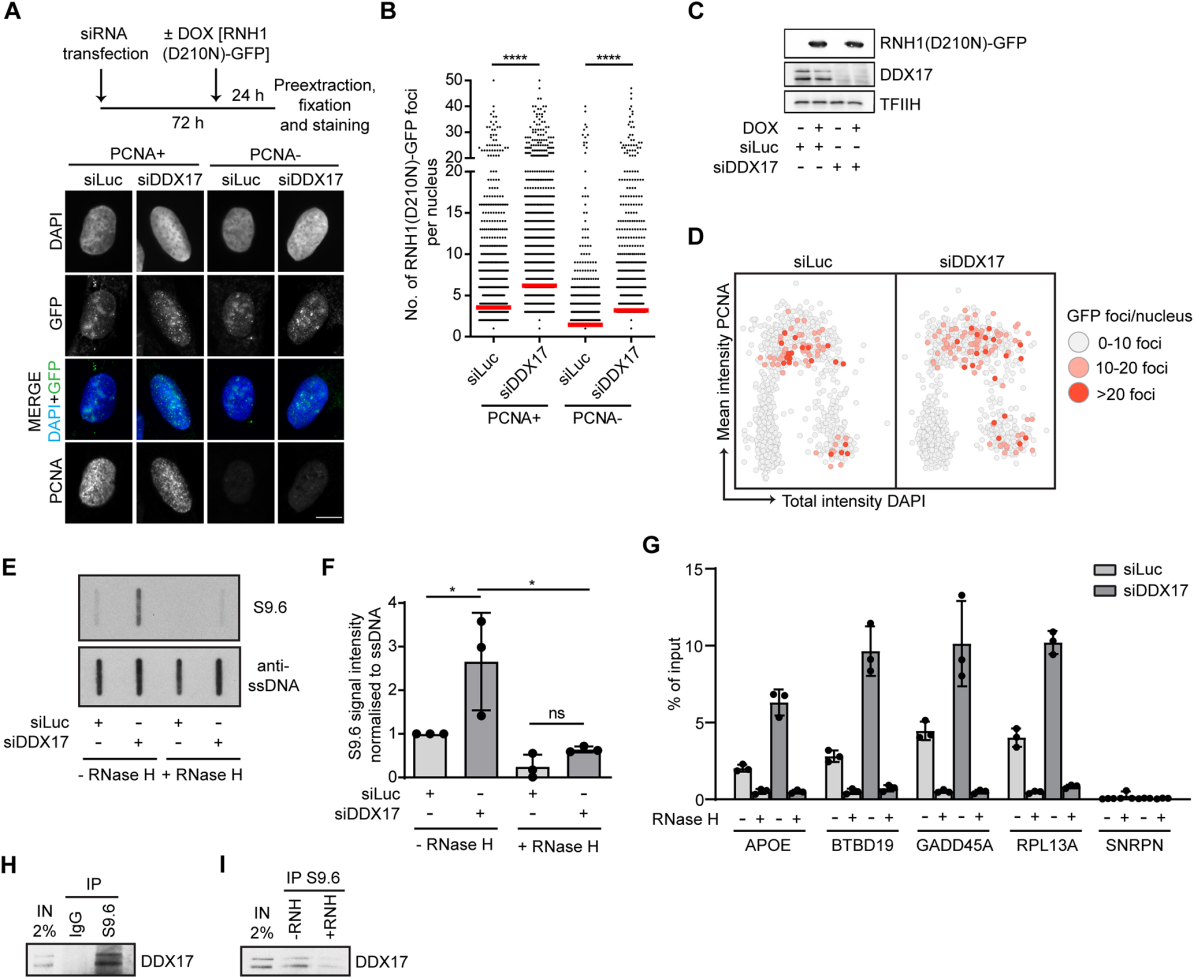
Loss of DDX17 induces accumulation of R-loops during S-phase. (**A**) Loss of DDX17 induces accumulation of R-loops. Top panel: schematic representation of experimental workflow. U-2 OS T-REx [RNH1(D210N)-GFP] cells grown on coverslips were transfected with indicated siRNAs for 72 h and treated with doxycycline (1 ng/ml) for last 24 h. Prior to fixation and staining with anti-PCNA antibody, cells were pre-extracted to eliminate unbound RNase H1. Bottom panel: Representative images. Nuclear foci of RNH1(D210N)-GFP indicate sites of R-loop formation. Scale bar, 10 μm. (**B**) Quantification of the number of RNH1(D210N)-GFP nuclear foci in PCNA-positive and PCNA-negative cells for indicated conditions (n ≥ 1700). Red horizontal lines indicate mean value. ****P ≤ 0.0001 (Mann–Whitney test). (**C**) Western blot analysis of extracts of U-2 OS T-REx [RNH1(D210N)-GFP] cells transfected with indicated siRNAs. (**D**) RNH1(D210N)-GFP foci accumulate in late S and G2 phase of the cell cycle in DDX17-depleted cells. Scatter plots of PCNA (y-axis) and DAPI (x-axis) intensities in individual DDX17-depleted (right) or mock-depleted (left) cells (*n* ∼ 450) to determine the cell cycle phase. The color code indicates the number of RNH1(D210N)-GFP foci per nucleus. (**E**) RNA:DNA slot blot analysis of genomic DNA isolated form U-2 OS cells transfected with indicated siRNAs. Genomic DNA was treated or not with RNase H. Blots were immunostained using S9.6 and anti-ssDNA antibodies. (**F**) Quantification of S9.6 signal on slot blots represented in (E). For each sample, S9.6 signal intensity was normalized to ssDNA intensity. The resulting values are normalized to the control siRNA (siLuc) and represent mean ± SD, *n* = 3. ns, non-significant (*P* > 0.05); * *P* ≤ 0.05 (One-way ANOVA). (**G**) Loss of DDX17 induces accumulation of R-loops at R-loop-prone loci in U-2 OS cells. DRIP-qPCR analyses were performed using S9.6 monoclonal antibody and genomic DNA isolated from U-2 OS cells transfected with indicated siRNAs. The R-loop-prone regions of the *APOE*, *BTBD19*, *GADD45A* and *RPL13A* genes were examined. An R-loop-free *SNRPN* gene locus was used as a negative control. Where indicated, the genomic DNA was treated with RNase H to remove RNA:DNA hybrids. Data are plotted as a percentage of the input, and represent the mean ± SD, *n* = 3. (**H,I**) DDX17 associates with R-loops. (**H**) U-2 OS cells were pre-extracted and sonicated to generate chromatin fragments with DNA size ranging from 800–200 bp. After centrifugation, sonicated cell lysates were immunoprecipitated with control IgG or S9.6 antibody followed by western blot analysis. (**I**) as in (H) except that, before immunoprecipitation, cell lysates were supplemented with 3 mM MgCl_2_ and incubated with RNaseH for 45 min at 37°C as indicated.

To further explore the role of DDX17 in the restart of R-loop-stalled forks, we investigated whether cells lacking DDX17 accumulate R-loops. For this, we made use of a previously established U-2 OS T-REx cell line conditionally expressing catalytically-inactive RNase H1 fused to green fluorescence protein [RNH1(D210N)-GFP], which serves as an R-loop reporter (35,36). DDX17- and mock-depleted cells expressing RNH1(D210N)-GFP for 24 h were subjected to QIBC analysis after pre-extraction and fixation. To determine DNA replication status of individual cells, immunofluorescence staining of PCNA was also carried out prior to the analysis. We observed that DDX17 depletion induced accumulation of RNH1(D210N)-GFP foci in nuclei of both PCNA-positive and PCNA-negative cells (Figure 3A-C), with the latter being exclusively in the G2-phase of the cell cycle (Figure 3D). Increased levels of R-loops in DDX17-depleted cells could be confirmed by slot blot analysis of isolated genomic DNA using S9.6 antibody, which recognizes RNA:DNA hybrids (Figure 3E, F). Moreover, by DRIP with S9.6 antibody followed by qPCR, we found that DDX17 depletion increased R-loop accumulation in several R-loop-prone genes, namely *APOE*, *BTBD19*, *GADD45A* and *RPL13A* (22,37,38) (Figure 3G). As a negative control, we used the *SNRPN* gene, which does not accumulate R-loops (38). As expected, DDX17 downregulation did not induce R-loop formation at this locus (Figure 3G). Importantly, the specificity of R-loop detection with this technique was further supported by the fact that *in vitro* treatment of genomic DNA with RNaseH prior to immunoprecipitation significantly reduced the R-loop signal for both DDX17-depleted and control (siLuc) cells (Figure 3G). Taken together, these data suggest that DDX17 prevents the accumulation of R-loops resulting from TRCs.

To demonstrate that DDX17 associates with R-loops, sonicated chromatin of U-2 OS cells was subjected to immunoprecipitation with S9.6 antibody followed by western blot analysis using DDX17 antibody. We found that DDX17 coimmunoprecipitated with S9.6 antibody but not with a control IgG in this assay (Figure 3H). Importantly, DDX17 coimmunoprecipitation was significantly reduced if the chromatin was pretreated with RNase H, indicating that it depends on the presence of RNA:DNA hybrids (Figure 3I). These data suggest that DDX17 acts directly on R-loops to promote the restart of R-loop stalled fork. This is also supported by the fact that DDX17 was identified in previous proteomic screens for proteins that associate with R-loops/RNA:DNA hybrids (28–31) (Supplementary Table S4).

**Figure 4.**
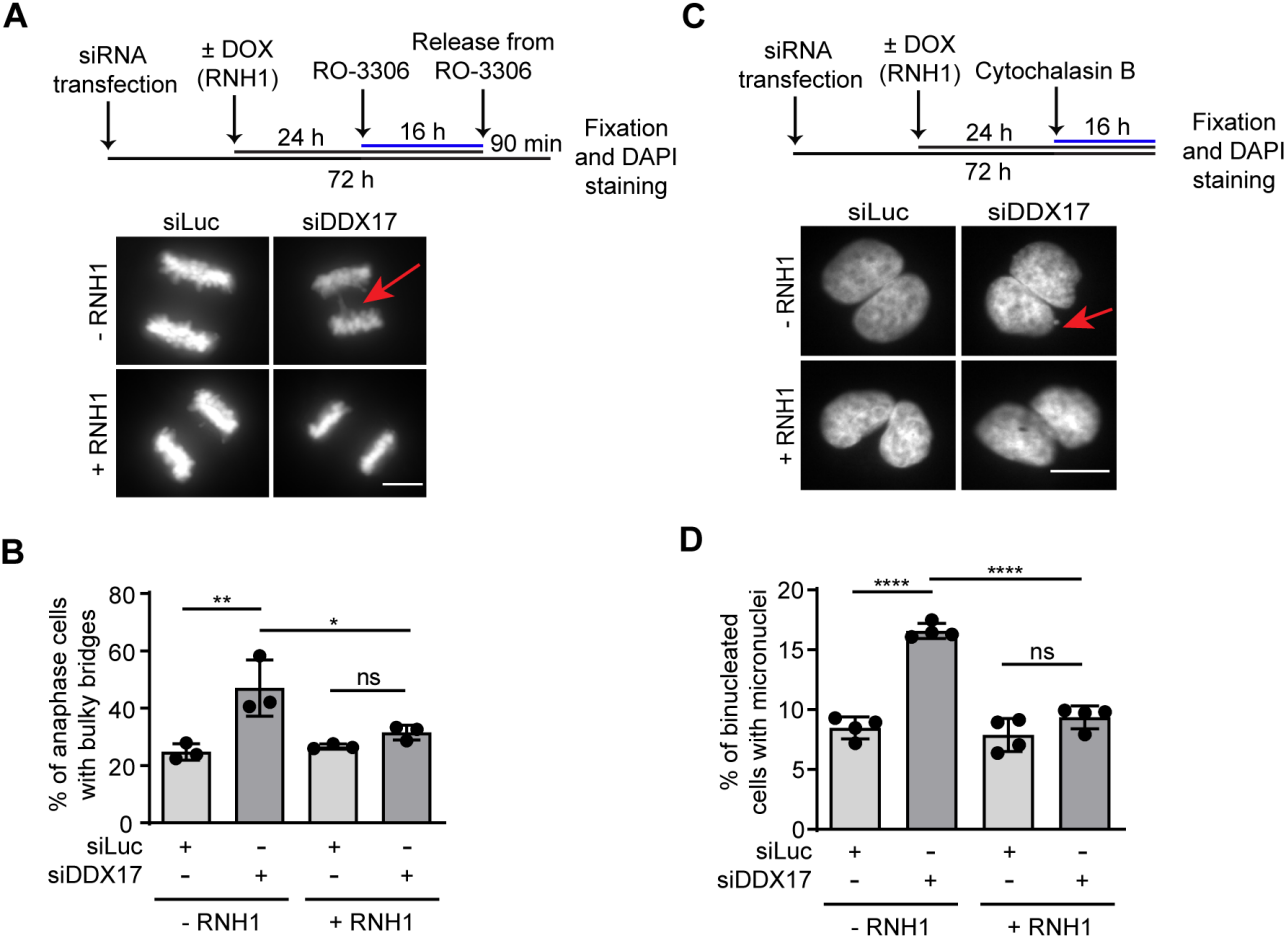
Loss of DDX17 induces the formation of R-loop-dependent anaphase bridges and micronuclei. (**A**, **B**) Loss of DDX17 induces formation of bulky anaphase bridges. (**A**) Top panel: Schematic representation of the experimental workflow of bulky anaphase bridge assay. U-2 OS T-REx [RNH1(WT)-GFP] cells were transfected with indicated siRNAs for 72 h and treated or not with doxycycline for the last 24 h to induce RNH1(WT)-GFP expression. Cells were synchronized at G2/M transition by treatment with RO-3306 for 16 h and subsequently released for 90 min into fresh medium, followed by fixation and DAPI staining. Bottom panel: Representative images of anaphase cells for indicated conditions. Red arrow points to a bulky anaphase bridge. Scale bar, 10 μm. (**B**) Quantitative analysis. Data are mean ± SD, *n* = 3. At least 25 anaphase cells were analyzed per experiment for each condition. ns, non-significant (*P* > 0.05); * *P* ≤ 0.05; ** *P* ≤ 0.01 (one-way ANOVA). (**C,D**) Loss of DDX17 induces formation of micronuclei. (**C**) Top panel: Schematic representation of the experimental workflow of micronucleus assay. U-2 OS T-REx [RNH1(WT)-GFP] cells were transfected with indicated siRNAs for 72 hours and treated or not with doxycycline for last 24 h. The last 16 h, cells were incubated with cytochalasin B (2 μg/ml) to inhibit cytokinesis, and subsequently fixed and stained with DAPI. Bottom panel: Representative images of binucleated cells for indicated conditions. Red arrow points to a micronucleus. Scale bar, 10 μm. (**D**) Quantitative analysis. Data are mean ± SD, *n* = 4. At least 150 binucleated cells were analyzed per experiment for each condition. ns, non-significant (*P* > 0.05); **** *P* ≤ 0.0001 (one-way ANOVA).

Persistent under-replicated DNA, resulting, for example, from unresolved TRCs, impairs chromosome segregation during mitosis (6,39–41). Therefore, we investigated whether DDX17-deficient cells show the mitotic phenotypes that arise due to chromosome segregation defects, namely anaphase bridges and micronuclei. In these experiments, we used the U-2 OS T-REx cell line inducibly expressing wild-type RNase H1 to assess dependence on R-loops. To monitor formation of bulky anaphase bridges, cells were treated with RO-3306, a selective CDK1 inhibitor, which reversibly arrests the proliferating cells at the G2/M transition point. Cell synchronization was followed by release into drug-free medium for 90 minutes and subsequent quantification of anaphase cells with or without DAPI-positive bulky bridges. We found that loss of DDX17 increased the frequency of bulky anaphase bridges in anaphase cells (Figure 4A, B). Importantly, this phenotype could be fully rescued upon overexpression of RNase H1 (Figure 4A, B). To measure the frequency of micronuclei, we treated the cells with cytochalasin B, which prevents cytokinesis, while leaving karyokinesis unaffected (42). We found that loss of DDX17 induced the formation of micronuclei, and that this was rescued by overexpression of RNase H1 (Figure 4C, D).

Taken together, our observations suggest that DDX17 might be involved in R-loop resolution at sites of TRCs, reducing replication stress and hence promoting faithful chromosome segregation.

### DDX17 acts in a common pathway with MUS81 to suppress R-loop-mediated replication stress

**Figure 5.**
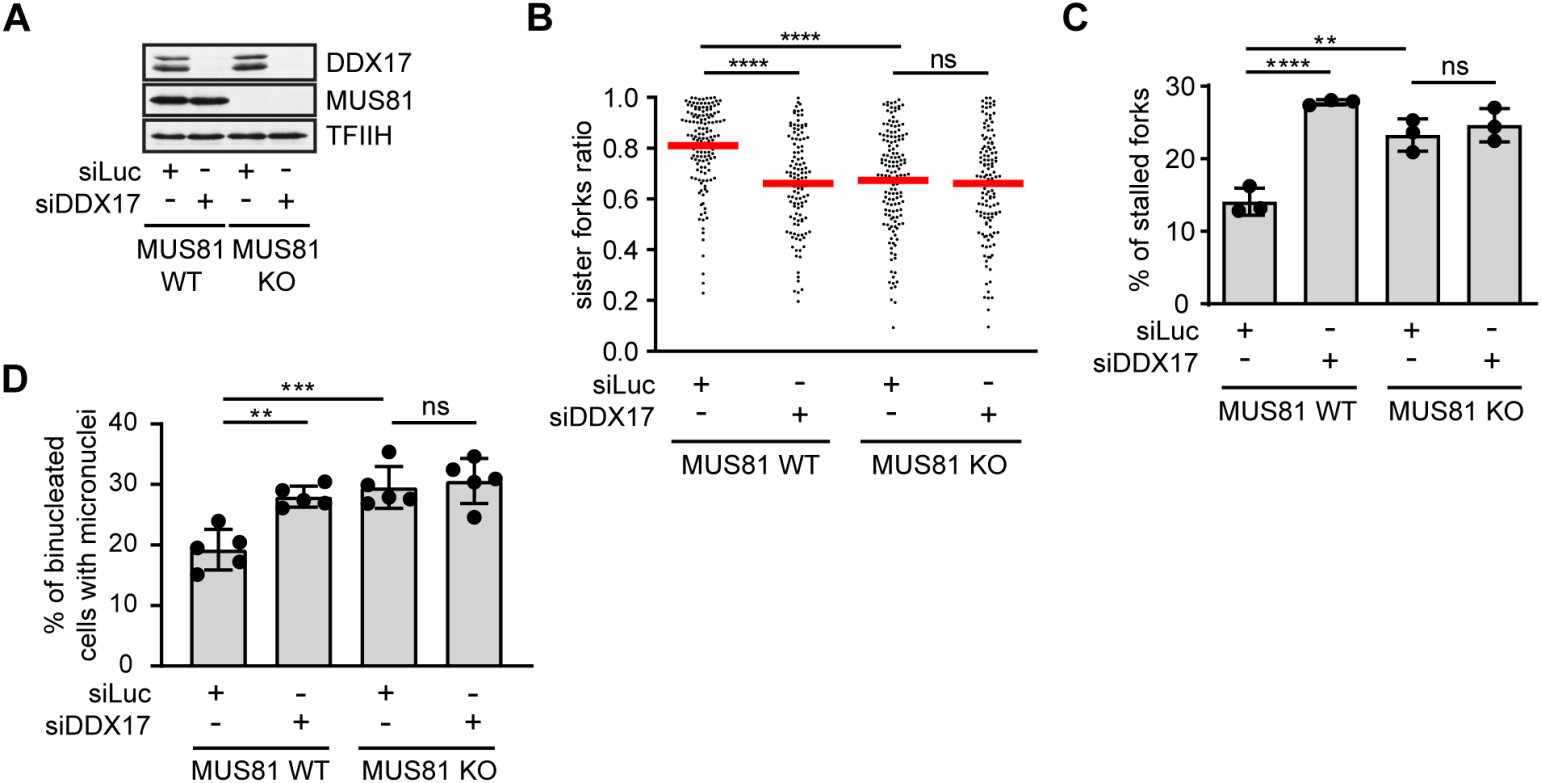
DDX17 acts in a common pathway with MUS81 to suppress R-loop-mediated replication stress. (**A**) Western blot analysis of the lysates of wild-type and MUS81 knockout HeLa Kyoto cells transfected with indicated siRNAs. (**B**) Scatter plot of the values of sister IdU tract length ratio (sister fork ratio; shorter IdU tract/longer IdU tract) measured for the indicated conditions (*n* ≥ 120). Red horizontal lines indicate mean value. ns, non-significant (*P* > 0.05); ****P ≤ 0.0001 (Mann–Whitney test). (**C**) Quantification of the replication fork stalling events (CldU tracts without an IdU tract) for indicated conditions. Data are mean ± SD, *n* = 3. ns, non-significant (*P* > 0.05); **P ≤ 0.01; ****P ≤ 0.0001 (one-way ANOVA). (**D**) Quantification of micronucleation events for indicated conditions. Data are mean ± SD, *n* = 5. ns, non-significant (*P* > 0.05); **P ≤ 0.01; ***P ≤ 0.001 (one-way ANOVA).

To assess whether DDX17 acts in the MUS81–LIG4–ELL pathway for restarting R-loop-stalled forks, we tested the effect of its siRNA-mediated depletion on the frequency of stalled replication forks and micronucleation in wild-type and MUS81 knockout HeLa Kyoto cells (6). By DNA fiber assay, we found that, like DDX17, MUS81 deficiency increased sister fork asymmetry and the percentage of CldU tracts without an IdU tract, phenotypes indicating replication fork stalling (Figure 5A-C). Moreover, MUS81 knockout cells showed elevated formation of micronuclei compared to the parental HeLa Kyoto cells (Figure 5D). Importantly, DDX17 depletion had no additional effect on the frequency of fork stalling and micronucleation in MUS81 knockout cells, although it increased these events in the parental HeLa Kyoto cells to levels similar to those caused by MUS81 knockout alone (Figure 5A–D). We could confirm this epistatic relationship also by co-depleting DDX17 and MUS81 in U2OS cells (Supplementary Figure S4). Taken together, these data suggest that DDX17 and MUS81 act in the same pathway to counteract R-loop-mediated fork stalling.

### DDX17 helicase unwinds R-loops *in vitro*

**Figure 6.**
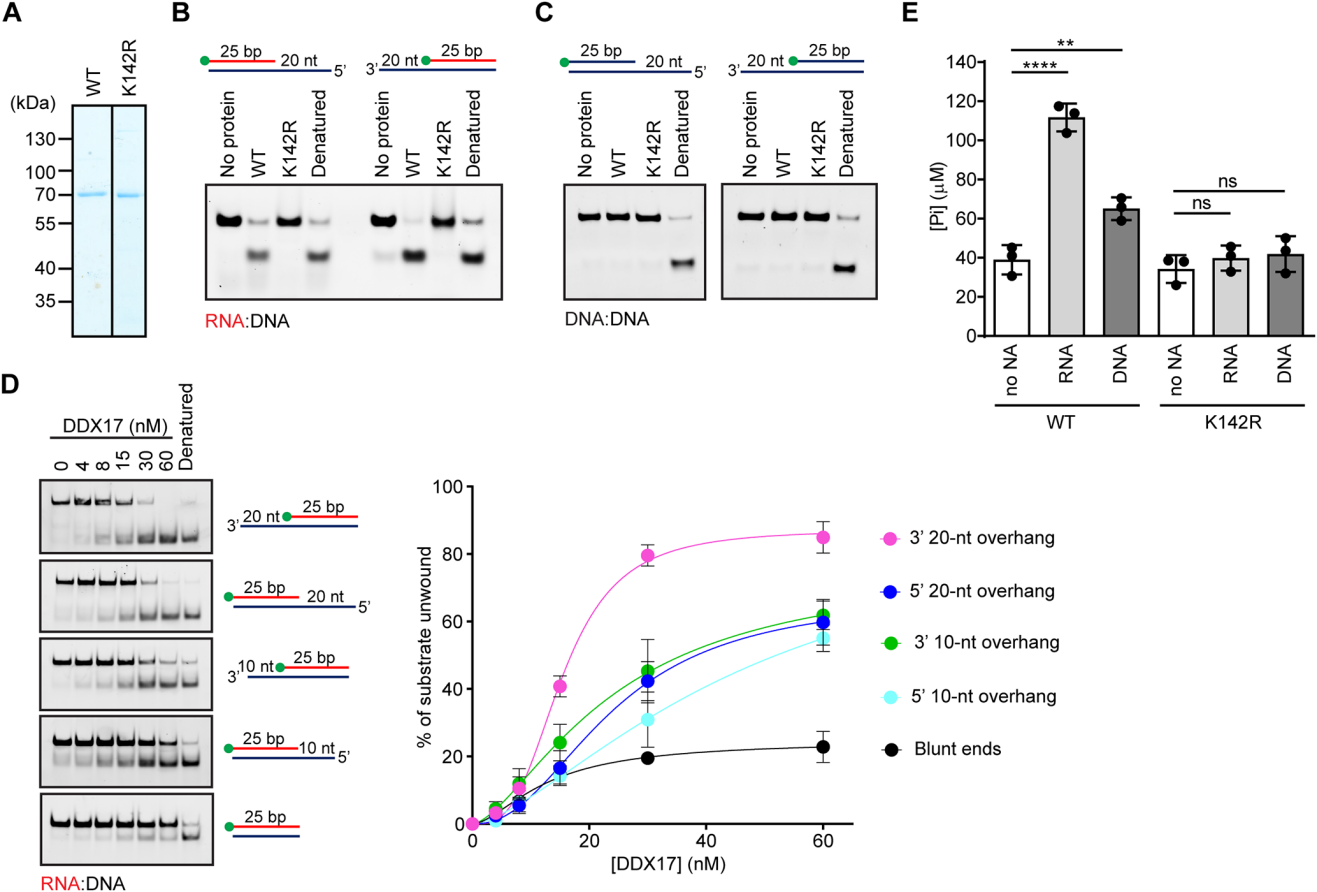
DDX17 helicase unwinds RNA:DNA hybrids *in vitro*. (**A**) Purified DDX17(WT) and DDX17(K142R) subjected to SDS-PAGE and Coomassie Blue staining. (B, C) DDX17 unwinds RNA:DNA duplexes but not DNA:DNA duplexes. 25-bp RNA:DNA (**B**) or DNA:DNA (**C**) duplexes with 3′ or 5′ 20-nt overhang were subjected to helicase assay. Reactions containing 5 nM oligonucleotide substrate and 60 nM DDX17, wild type (WT) or K142R mutant, were carried out at 37°C for 20 min. Reactions were initiated by adding ATP to a final concentration of 5 mM. Reaction products were analyzed on native polyacrylamide gels. Schemes of oligonucleotide substrates are shown on the top. The position of FAM label is indicated (green ball). Lane 1, reaction without protein; lane 4, heat denatured substrate. (**D**) Unwinding of 25-bp RNA:DNA duplexes with overhangs of various lengths (5 nM) were incubated with increasing concentrations of DDX17 (0–60 nM). Unwinding reactions were carried at 37°C for 20 min and analyzed as in (B). Left panel: Representative images of gels. Right panel: Quantification of gels represented in the left panel. Data are mean ± SD, *n* = 3. (**E**) ATPase activity of DDX17 in the presence of RNA or DNA oligonucleotides, or without any nucleic acid (no NA). Reactions containing 1 nM oligonucleotide, 40 nM DDX17 (WT or K142R) and 2 mM ATP were carried out at 37°C for 60 min. The concentration of inorganic phosphate released by ATP hydrolysis was estimated by malachite green assay. Data are mean ± SD, *n* = 3. ns, non-significant (p > 0.05); **P ≤ 0.01; ****P ≤ 0.0001 (one-way ANOVA).

DDX17 has been described as a DEAD-box RNA helicase existing in two isoforms: p72 and p82, which result from alternative translation initiation sites (43). DDX17 possesses RNA-dependent ATPase activity (14) and is capable of unwinding RNA:RNA duplexes but not DNA:DNA duplexes (44). To explore the hypothesis that DDX17 is involved in R-loop resolution, we first tested whether DDX17 can unwind RNA:DNA hybrids. For this, we produced the 72 kDa isoform of DDX17 in *E. coli* and purified it to apparent homogeneity (Figure 6A). Moreover, we prepared a DDX17 mutant carrying a K142R substitution in helicase motif I, which is required for the ATPase and helicase activities of the enzyme (45). Purified recombinant proteins were subjected to helicase assays with a fluorophore (5′-FAM)-conjugated RNA:DNA or DNA:DNA duplexes (25 bp in length) containing either 3′ or 5′ ssDNA overhangs of 20 nucleotides (nt) in length. We observed that wild type DDX17 could unwind both RNA:DNA substrates, but not the DNA:DNA substrates (Figure 6B, C). This RNA/DNA helicase activity of DDX17 was fully dependent upon the presence of ATP (Supplementary Figure S5) and was not seen with the DDX17(K142R) mutant (Figure 6B). Moreover, a protein titration experiment with 25-bp RNA:DNA duplex substrates containing ssDNA tails of varying lengths (0, 10 and 20 nt) revealed that DDX17 requires ssDNA tail for efficient RNA:DNA duplex unwinding being most efficient on 3′-tailed substrates (Figure 6D). This suggests that DDX17 can unwind RNA:DNA hybrids by translocation on DNA in 3′-to-5′ direction. In support of this notion, we found that ATP hydrolysis by DDX17 was stimulated not only by ssRNA but also ssDNA (Figure 6E), suggesting that DDX17 is capable of translocating along both RNA and DNA strands. The seemingly dual polarity of DDX17 helicase, which was also observed on partial RNA:RNA duplexes (44), could be explained by an assumption that on 5′-ssDNA-tailed RNA:DNA duplexes (or 5′-tailed RNA:RNA duplex), the helicase is loaded on the ssDNA (ssRNA) tail but then translocates along the RNA strand in 3′-to-5′ direction to mediate duplex unwinding. Absence of such 5′-tail would reduce the ability of the helicase to initiate the 3′-5′ translocation.

**Figure 7.**
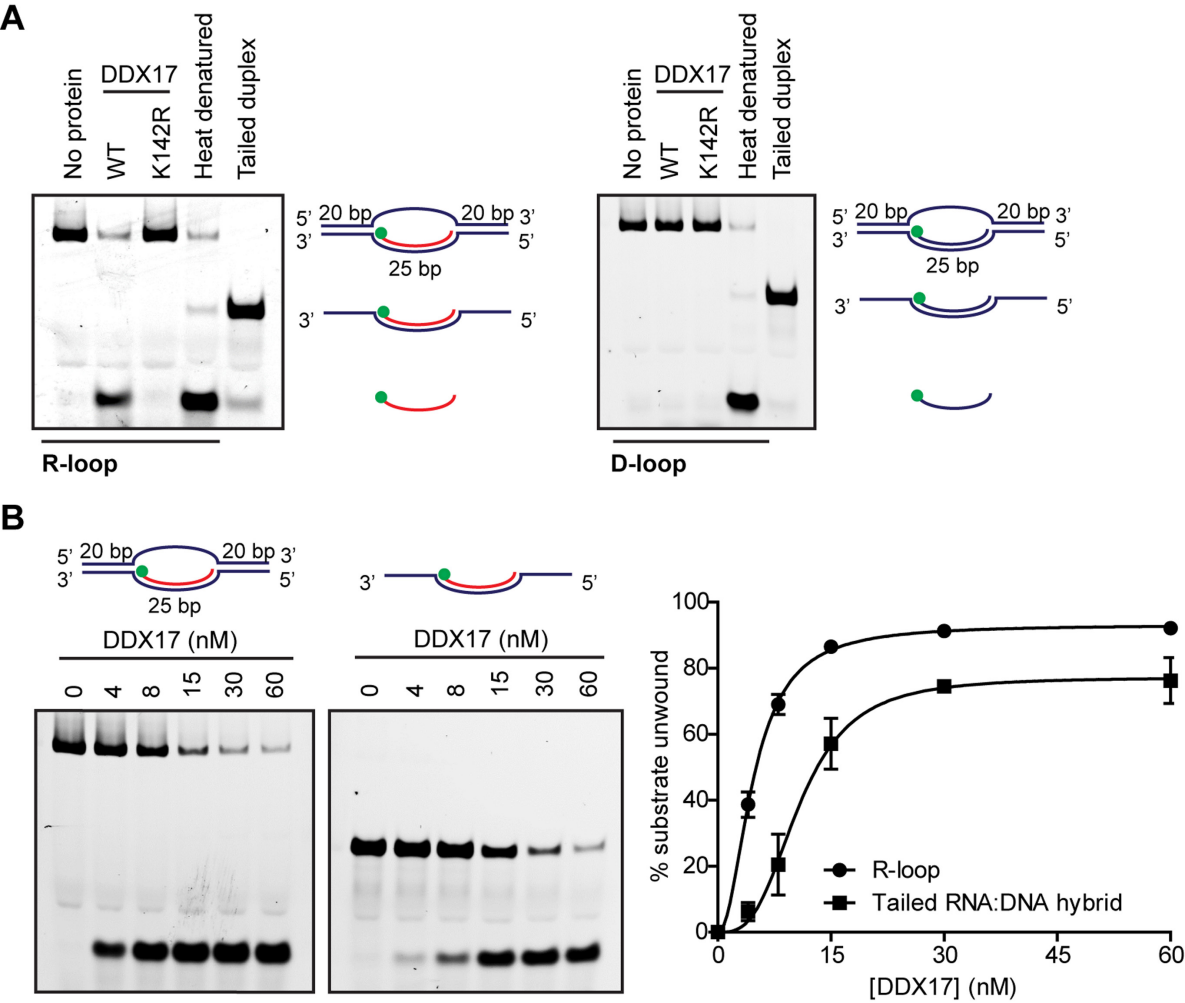
DDX17 helicase preferentially unwinds R-loops *in vitro*. (**A**) Purified DDX17 unwinds R-loop but not D-loop structures. 5 nM R-loop (left panel) and D-loop (right panel) substrates were incubated with 60 nM wild-type (WT) or K142R mutant of DDX17 at 37°C for 10 min. Reactions were initiated by adding ATP to a final concentration of 5 mM. Reaction products were analysed on native polyacrylamide gels. Schemes of substrates are indicated next to the bands. The position of FAM label is indicated as a green ball in the illustration. Lane 1, reaction without protein; lane 4, heat denatured substrate; lane 5, tailed duplex used as a marker. (**B**) Unwinding of R-loop and ssDNA-tailed RNA:DNA hybrid by DDX17 helicase. 5 nM R-loop or ssDNA-tailed RNA:DNA hybrid substrates were incubated with increasing concentrations of DDX17 helicase (0–60 nM) as indicated. Unwinding reactions were initiated by adding ATP to a final concentration of 5 mM and carried out at 37°C for 10 min. Reaction products were analysed as in (A). Right panel: Quantification of gels represented in the left panel. Data are mean ± SD, *n* = 3.

To test whether DDX17 unwinds R-loops, we prepared an oligonucleotide-based R-loop structure consisting of a 25-bp RNA:DNA hybrid and a ssDNA loop of 25 Ts flanked by 20-bp DNA duplexes (the 5′-FAM-labeled RNA oligonucleotide in this structure has the same sequence as the above RNA:DNA hybrids). We found that DDX17 could efficiently unwind the RNA:DNA hybrid in this structure in a manner dependent on its ATPase activity while it did not show any helicase activity on the corresponding D-loop structure (Figure 7A). Notably, DDX17 unwound the R-loop substrate more efficiently than the ssDNA-tailed RNA:DNA hybrid derived from this structure (Figure 7B). These data indicate that DDX17 has a preference for unwinding R-loop structures.

### Helicase activity of DDX17 is required for the restart of R-loop-stalled forks

**Figure 8.**
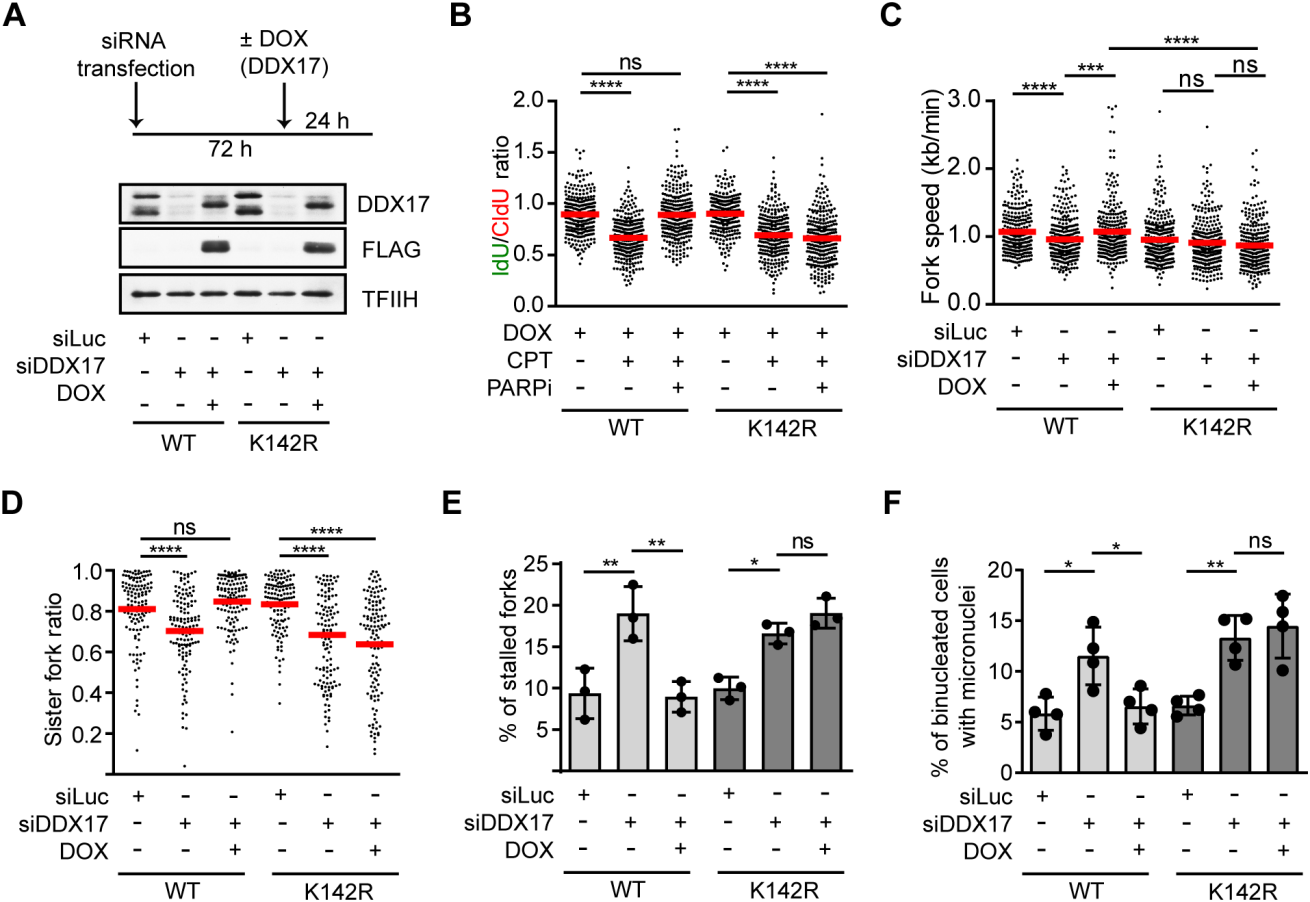
Helicase activity of DDX17 is required for the restart of R-loop-stalled forks. (**A**) Top panel: Experimental workflow. U-2 OS T-REx cells carrying DDX17(WT)-FLAG or DDX17(K142R)-FLAG transgenes were transfected with siRNA targeting endogenous DDX17 for 72 h. After 48 h, cells were treated with doxycycline to induce expression of the transgene. Bottom panel: Western blot analysis of extracts of the above cells. (**B**) PARP inhibition does not rescue CPT-induced replication fork slowing in cells expressing DDX17(K142R). Scatter plot of the values of IdU/CldU tract length ratio obtained for indicated conditions is shown (n ≥ 300). Red horizontal lines indicate mean value. ns, non-significant (*P* > 0.05); ****P ≤ 0.0001 (Mann–Whitney test). (**C**) Cells expressing DDX17(K142R) display a reduced replication fork velocity. Scatter plot of the values of velocity (kb/min) of individual forks obtained for indicated conditions is shown (n ≥ 300). Experimental workflow of DNA fiber assay used is shown in Figure 2H. Red horizontal lines indicate mean value. ns, non-significant (*P* > 0.05); ***P ≤ 0.001; ****P ≤ 0.0001 (Mann–Whitney test). (**D**) Cells expressing DDX17(K142R) display sister fork asymmetry. Scatter plot of the values of sister IdU tract length ratio (sister fork ratio; shorter IdU tract/longer IdU tract) measured for the indicated conditions is shown (n ≥ 124). Red horizontal lines indicate mean value. ns, non-significant (*P* > 0.05); ****P < 0.0001 (Mann–Whitney test). (**E**) Cells expressing DDX17(K142R) show increased frequency of stalled replication forks determined as in Figure 2J. Data are mean ± SD, *n* = 3. ns, non-significant (*P* > 0.05); *P ≤ 0.05; **P ≤ 0.01 (One-way ANOVA). (**F**) Cells expressing DDX17(K142R) show increased micronucleation after mitosis determined as in Figure 4C,D. Data are mean ± SD, *n* = 3. ns, non-significant (*P* > 0.05); * *P* ≤ 0.05; **P ≤ 0.01 (one-way ANOVA).

To this end, we have shown that DDX17 is necessary for the restart of R-loop-stalled replication forks and unwinds RNA:DNA hybrids *in vitro*. To test whether DDX17 promotes the restart of R-loop-stalled replication forks in a manner dependent on its helicase activity, we generated U-2 OS T-REx cell lines expressing siRNA-resistant versions of either DDX17(WT)-FLAG (wild type form of DDX17) or DDX17(K142R)-FLAG (DDX17 K142R mutant) transgenes from a doxycycline-regulated promoter. We could confirm that expression of both recombinant proteins in stably transfected clones occurred only in cell nuclei after the addition of doxycycline (Figure 8A; Supplementary Figure S6). Importantly, we found that cells expressing DDX17(K142R)-FLAG in absence of endogenous DDX17 failed to restart CPT-stalled forks upon the boost with PARPi (Figure 8B). On the contrary, ectopic expression of DDX17(WT)-FLAG restored unrestrained DNA synthesis in DDX17-depleted cells treated with CPT and PARPi (Figure 8B). As expected, DDX17 depletion in both clones tested also reduced replication velocity and increased the frequency of fork stalling events and micronucleation in unchallenged condition (Figure 8C–F). These phenotypes could be again rescued by expression of DDX17(WT)-FLAG but not by expression of DDX17(K142R)-FLAG (Figure 8C–F). These results suggest that DDX17 helicase activity is necessary for the restart of R-loop-stalled replication forks.

## DISCUSSION

Accumulating evidence suggests that impairment of replication fork progression by head-on coming transcription complexes is caused by the formation of co-transcriptional R-loops (4,5). Our recent work has suggested that the replication forks stalled by R-loops can be restarted by a mechanism involving fork cleavage by MUS81 endonuclease and subsequent fork re-ligation by the LIG4/XRCC4 complex, which allows reactivation of transcription and passage of the transcription complex across the fork junction to the lagging arm (6). Here, we identify the DEAD-box helicase DDX17 as a new component of this pathway acting through its helicase activity. Given our findings that DDX17 associates with chromatin in a manner dependent on the presence of RNA:DNA hybrids (Figure 3H, I) and has the ability to efficiently unwind R-loops *in vitro* (Figure 7), it is tempting to speculate that DDX17 is involved in R-loop unwinding at the sites of TRCs, which is a prerequisite for the putative transcription restart and the subsequent progression of the reactivated replication fork (6). Consistently, we found that loss of DDX17 induced the accumulation of R-loops in S and G2 cells (Figure 3A-D), which is likely to prevent the completion of DNA replication and thus impair chromosome segregation during mitosis. In agreement with this assumption, we found that DDX17 helicase deficiency increased the frequency of R-loop-dependent anaphase bridges and micronuclei (Figure 4), indicating a chromosome segregation defect.

There is growing evidence that DEAD-box RNA helicases play important roles in the maintenance of genome integrity in mammalian cells (46). Interestingly, in addition to DDX17, also loss of other DEAD-box helicases, specifically DDX5 (47,48), DDX19 (49), DDX21 (50), DDX39B (51) and DDX41 (31), leads to accumulation of RNA:DNA hybrids in human cells. Moreover, DDX3X (52), DDX19 (49), DDX21 (50), DDX39B (51) and DDX41 (31) have been reported to unwind RNA:DNA hybrids *in vitro*. In this regard, it is intriguing that depletion of one particular RNA/DNA helicase, DDX17, can eliminate R-loop processing during TRC resolution. The possible explanations could be that DDX17 possesses a unique activity required for disruption of R-loop-forming transcription complexes or that DDX17 might be specifically recruited to the sites of TRCs *via* binding to other proteins to mediate R-loop clearance. We cannot also exclude the possibility that multiple RNA/DNA helicases act in a complementary fashion to eliminate R-loops at sites of R-loop-mediated TRCs to mediate replication restart. In fact, cells lacking DDX19 have been shown to display R-loop-dependent replication stress (49). Moreover, elevated formation of transcription-dependent micronuclei has been observed in human cells deficient in the putative RNA/DNA helicase Senataxin (22). Senataxin deficiency was also found to cause increased transcription stress near promoters that correlated with high GC skew and R-loop accumulation at promoter-proximal regions, a phenotype that may result due to defective TRC resolution (22). Consistent with this notion, the budding yeast homolog of Senataxin, Sen1, has been shown to promote replication fork progression across RNAPII-transcribed genes by counteracting RNA:DNA hybrids (53). Thus, further studies are needed to fully understand the molecular mechanisms underlying the resolution of R-loop-mediated TRCs in human cells.

## DATA AVAILABILITY

Primary data are available in this manuscript or as Supplementary Data. The mass spectrometry proteomics data have been deposited to the ProteomeXchange Consortium via the PRIDE partner repository with the dataset identifier PXD034331 and 10.6019/PXD034331.

## Supplementary Material

gkac1116_Supplemental_Files

## Notes

Present address: Zuzana Nascakova and Martin Andrs, Institute of Molecular Cancer Research, University of Zurich, Winterthurerstrasse 190, 8057 Zurich, Switzerland.

Present address: Zdenka Hasanova, CEITEC, Masaryk University, Kamenice 5, 62500 Brno, Czech Republic.
