# Supplementary material for: DDX17 helicase promotes resolution of R-loop-mediated transcription–replication conflicts in human cells
Source: Nucleic Acids Res. 2022 Dec 1;50(21):12274–90. doi: 10.1093/nar/gkac1116 (PMC9757067; doi:10.1093/nar/gkac1116)
Supplement: gkac1116_Supplemental_Files [file gkac1116_supplemental_files.zip › Supplementary data.pdf]

Supplementary Table S1. DNA and RNA oligonucleotides and schemes of RNA/DNA substrates used in this study.

Name	Length	Sequence (5' → 3')
A	25	(FAM) - AGCUAUGACCAUGAUUACGAAUUGC
B	25	(FAM) - AGCTATGACCATGATTACGAATTGC
C	25	GCAATTCGTAATCATGGTCATAGCT
D	35	TTTTTTTTTTGCAATTCGTAATCATGGTCATAGCT
E	35	GCAATTCGTAATCATGGTCATAGCTTTTTTTTTTT
F	45	GCAATTCGTAATCATGGTCATAGCTTTTTTTTTTTTTTTTTTTTT
G	45	TTTTTTTTTTTTTTTTTTTTGCAATTCGTAATCATGGTCATAGCT
H	45	GCAAUUCGUAAUCAUGGUCAUAGCUUUUUUUUUUUUUUUUUUUUU
I	65	GGGTGAACCTGCAGGTGGGCTTTTTTTTTTTTTTTTTTTTTTTTTTGGTAGAA TTCGGCAGCGTC
J	65	GACGCTGCCGAATTCTACCAGCAATTCGTAATCATGGTCATAGCTGCCCAC CTGCAGGTTCACCC

Name	Scheme	Name	Scheme
Blunt ended RNA:DNA hybrid	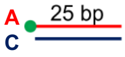	DNA:DNA duplex with 20-nt 3'-overhang	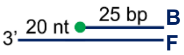
RNA:DNA hybrid with 10-nt 5'-overhang	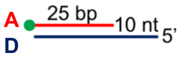	R-loop	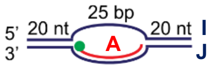
RNA:DNA hybrid with 10-nt 3'-overhang	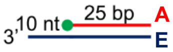	D-loop	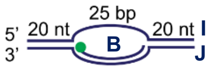
RNA:DNA hybrid with 20-nt 5'-overhang	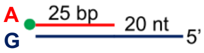	RNA:DNA hybrid with 20-nt ssDNA tails	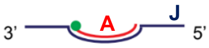
RNA:DNA hybrid with 20-nt 3'-overhang	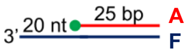	DNA:DNA duplex with 20-nt ssDNA tails	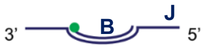
DNA:DNA duplex with 20-nt 5'-overhang	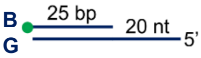		

Supplementary Table S2. List of human proteins identified by BioID-based proteomic analysis of R-loop interactome.

Supplementary Table S3. List of significantly enriched proteins.

Condition	Identified proteins
DMSO or CPT	AHCTF1, ANLN, CCT6A, CHAMP1, COIL, DDX3X, DDX42, FUS, IK, KIF23, KIF4A, MKI67, NAT10, NCL, NOL6, NOLC1, NONO, NPM1, PSPC1, RBM17, RBM25, RBM27, RIF1, RNASEH1, SART1, SF3B1, SF3B2, SNW1, TMPO, TPX2, WAPAL, ZC3H18
CPT	ACTN4, AHCTF1, ANLN, CALR, CCT6A, CHAMP1, CLTC, COIL, CORO1C, CSRP1, DDX17, DDX3X, DDX42, DDX5, DHX15, DHX9, EEF1A1P5, EEF1B2, EEF1D, EEF1G, EIF4A1, FLNA, FLNC, FUS, FXR1, GPKOW, HADHB, HIST2H2BE, HNRNPA1, HNRNPA2B1, HNRNPA3, HNRNPD, HNRNPF, HNRNPH1, HNRNPH3, HNRNPK, HNRNPL, HNRNPM, HNRNPR, HNRNPU, HSPA9, IK, INTS12, KIF23, KIF4A, MATR3, MGA, MKI67, NAT10, NCL, NOL6, NOLC1, NONO, NPM1, PABPC1, PDIA6, PHB, PHB2, PLEC, PPP1CC, PRKDC, PSPC1, RBM17, RBM25, RBM27, RBMX, RIF1, RNASEH1, RPL12, RPL9, RPLP0, RPN1, RPS2, RPS3, RPS7, RPSA, SART1, SEPT9, SERBP1, SF3A1, SF3B1, SF3B2, SFPQ, SND1, SNRNP200, SNW1, SYNCRIP, TARDBP, THRAP3, TMPO, TPX2, TUFM, UBAP2L, VDAC2, VIM, WAPAL, WBP11, XRCC6, ZC3H18

Supplementary Table S4. Overlap between proteins identified in this study and previously published RNA:DNA hybrid interactomes.

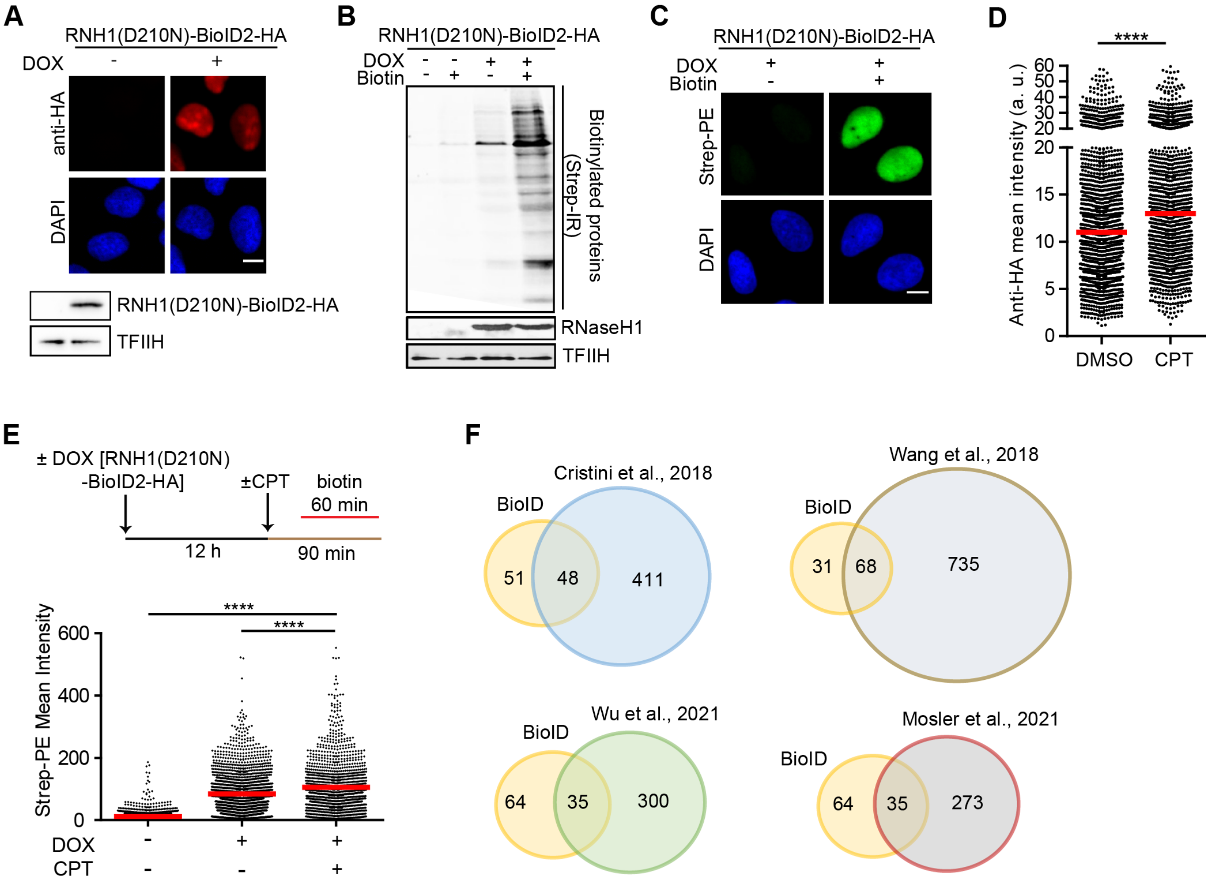

Supplementary Figure S1. BioID-based proteomic analysis of R-loop interactome at sites of transcription-replication conflicts.

(**A**) Expression of RNH1(D210N)-BioID2-HA in U-2 OS T-REx [RNH1(D210N)-BioID2-HA] treated or not with doxycycline (1 ng/ml) for 24 h. Expression of RNH1(D210N)-BioID2-HA was assessed by western blot using anti-RNaseH1 antibody (bottom panel), and by immunofluorescence microscopy using anti-HA antibody (top panel). Nuclei were counterstained with DAPI. Scale bar, 10 µm.

(**B,C**) In U-2 OS T-REx cells expressing [RNH1(D210N)-BioID2-HA] protein biotinylation occurs upon biotin addition. Cells were treated or not with doxycycline (1 ng/ml) and 50 µM biotin for 24 h. (**B**) Cell extracts were subjected to western blot analysis. Protein biotinylation was detected using streptavidin labelled with an infrared dye. (**C**) Cells were fixed and subjected to staining with streptavidin-phycoerythrin conjugate. Nuclei were counterstained with DAPI. Scale bar, 10 µm.

(**D**) CPT treatment increases chromatin binding of RNH1(D210N)-BioID2-HA in U-2 OS T-REx [RNH1(D210N)-BioID2-HA] cells. Cells were treated with doxycycline (1 ng/ml) for 24 h and with 100 nM CPT or DMSO for the last 90 min. After preextraction and fixation, cells we subjected to immunofluorescence staining with anti-HA antibody followed by QIBC analysis. ****$p \leq 0.0001$ (Mann-Whitney test).

(**E**) CPT treatment increases biotinylation in nuclei of U-2 OS T-REx [RNH1(D210N)-BioID2-HA] cells. Top panel: Experimental workflow. The expression of RNH1(D210N)-BioID2-HA fusion protein was induced by addition of doxycycline at a concentration of 0,2 ng/ml for 12 h. Cells were exposed to CPT (100 nM) or treated with vehicle alone (DMSO) for the last 90 min. Biotin (50 µM) was added into the

cell culture medium for the last 60 min of the incubation time. Bottom panel: Quantification of the strep-PE intensity in the cell nucleus. Scatter plot of the values of the strep-PE intensities obtained for indicated conditions is shown ($n \geq 1400$). Red horizontal lines indicate mean value. ****$p \leq 0.0001$ (Mann-Whitney test).

(**F**) Venn diagrams showing overlap between proteins identified in this study and previously published RNA:DNA hybrid interactomes as indicated.

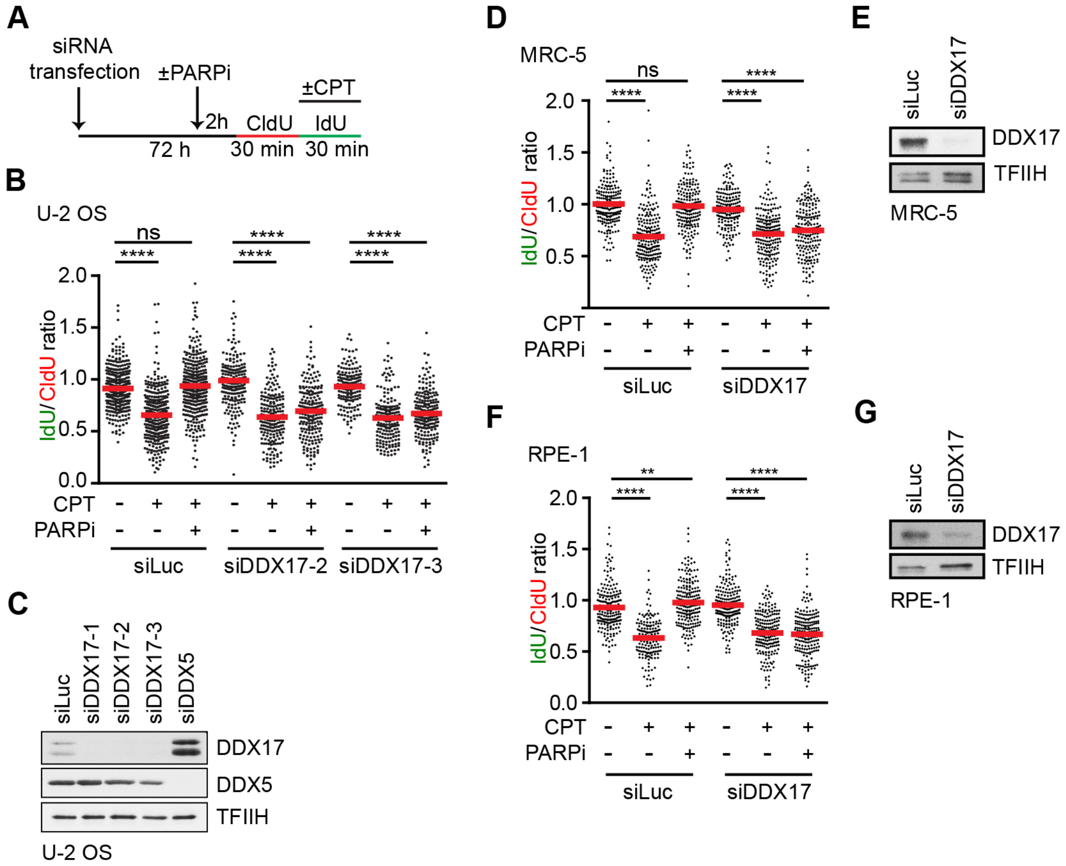

Supplementary Figure S2. DDX17 promotes replication restart following R-loop-mediated fork stalling. (**A**) Experimental workflow of DNA fiber assays for (B), (D) and (F). Cells were transfected with indicated siRNAs for 72 hours followed by pulse-labelling with CldU and IdU. During IdU labelling, cells were treated with 100 nM CPT or DMSO. Where indicated, 2 h before the labelling and during the labeling, cells were supplemented with 10 μM PARP inhibitor olaparib (PARPi).

(**B**) Effect of DDX17 depletion on the rescue of CPT-induced replication fork slowing by PARP inhibition (PARPi) in U-2 OS cells. Scatter plot of the values of IdU/CldU tract length ratio obtained for indicated conditions is shown (n ≥ 300). Red horizontal lines indicate mean value. ns, non-significant ($p > 0.05$); ****$p \leq 0.0001$ (Mann-Whitney test).

(**C**) Western blot analysis of extracts of U-2 OS cells transfected with indicated siRNAs.

(**D,F**) Effect of DDX17 depletion on the rescue of CPT-induced replication fork slowing by PARPi in MRC-5 (**D**) and RPE-1 (**F**) cells. Scatter plot of the values of IdU/CldU tract length ratio obtained for indicated conditions is shown (n ≥ 200). Red horizontal lines indicate mean value. ns, non-significant ($p > 0.05$); **$p \leq 0.01$; ****$p \leq 0.0001$ (Mann-Whitney test).

(**E,G**) Western blot analysis of extracts of MRC-5 (**E**) and RPE-1 (**G**) cells transfected with indicated siRNAs.

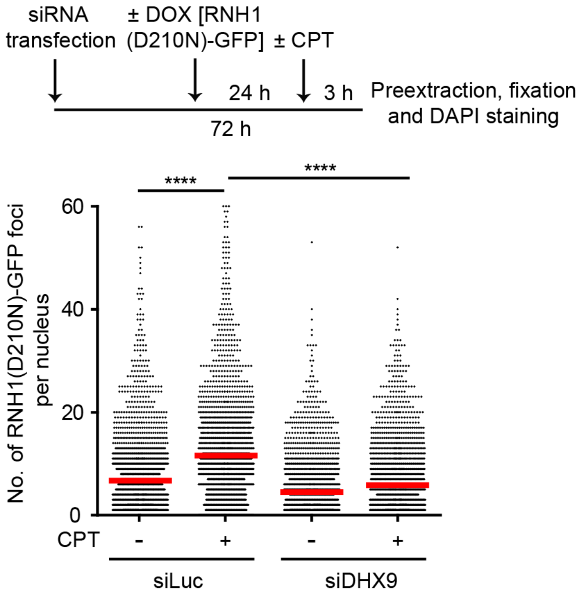

Supplementary Figure S3. Loss of DHX9 compromises R-loops formation upon CPT treatment.
Top panel: schematic representation of experimental workflow. U-2 OS T-REx [RNH1(D210N)-GFP] cells grown on coverslips were transfected with indicated siRNAs for 72 h and treated with doxycycline (1 ng/ml) for 24 h. For the last 3 h, cells were treated with 100 nM CPT. Prior to fixation, cells were pre-extracted to eliminate unbound RNase H1. Bottom panel: Quantification of the number of RNH1(D210N)-GFP nuclear foci for indicated conditions ($n \geq 2210$). Red horizontal lines indicate mean value. ****$p \leq 0.0001$ (Mann-Whitney test).

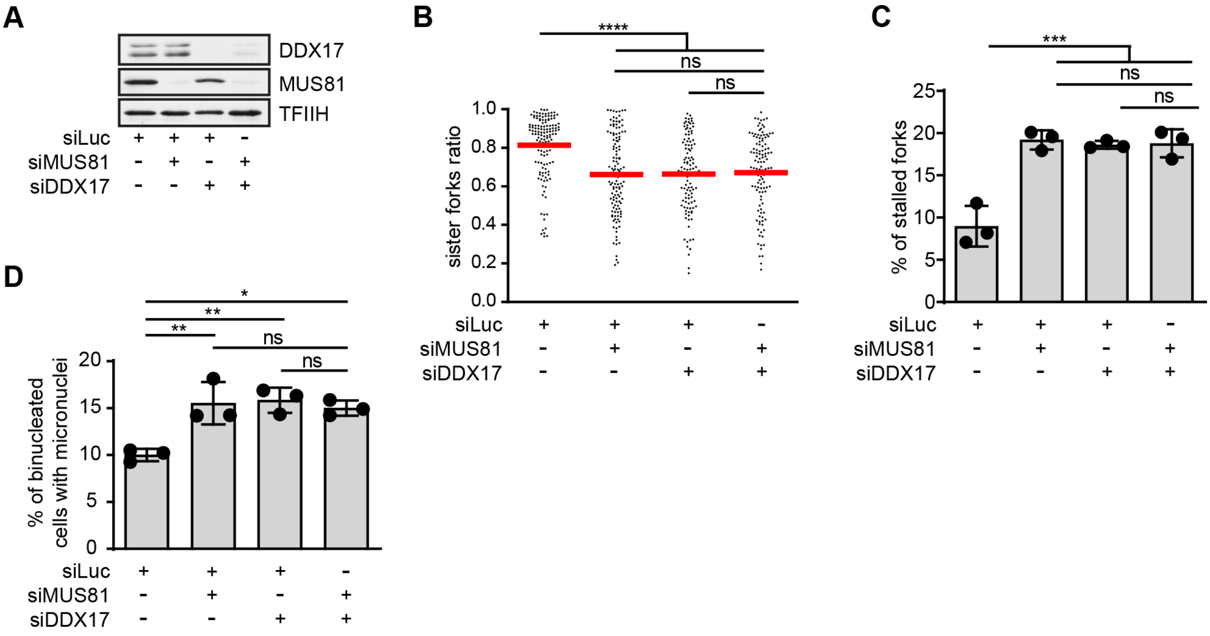

Supplementary Figure S4. DDX17 acts in a common pathway with MUS81 to suppress R-loop-mediated replication stress.

(**A**) Western blot analysis of the lysates of U-2 OS cells transfected with indicated siRNAs.

(**B**) Scatter plot of the values of sister IdU tract length ratio (sister fork ratio; shorter IdU tract/longer IdU tract) measured for the indicated conditions ($n \geq 110$). Red horizontal lines indicate mean value. ns, non- significant ($p > 0.05$); ****$p \leq 0.0001$ (Mann-Whitney test).

(**C**) Quantification of the replication fork stalling events (CldU tracts without an IdU tract) for indicated conditions. Data are mean ± SD, n = 3. ns, non-significant ($p > 0.05$); ***$p \leq 0.001$ (one-way ANOVA test).

(**D**) Quantification of micronucleation events for indicated conditions. Data are mean ± SD, n = 3. ns, non-significant ($p > 0.05$); *$p \leq 0.05$; **$p \leq 0.01$ (one-way ANOVA test).

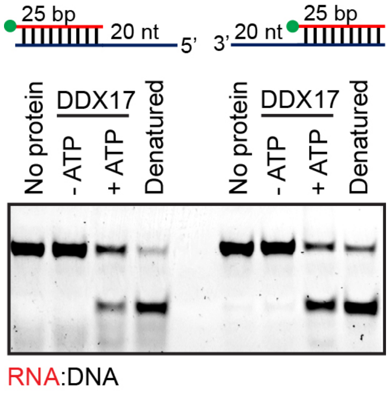

Supplementary Figure S5. Unwinding of RNA:DNA hybrids by DDX17 is ATP dependent.
5 nM RNA:DNA duplexes with 3' or 5' 20-nt overhang were incubated with 60 nM DDX17 in the absence or presence of 5 mM ATP. Reactions were carried out at 37°C for 10 min. Reaction products were analyzed on native polyacrylamide gels. Lane 1, reaction without protein; lane 4, heat denatured substrate.

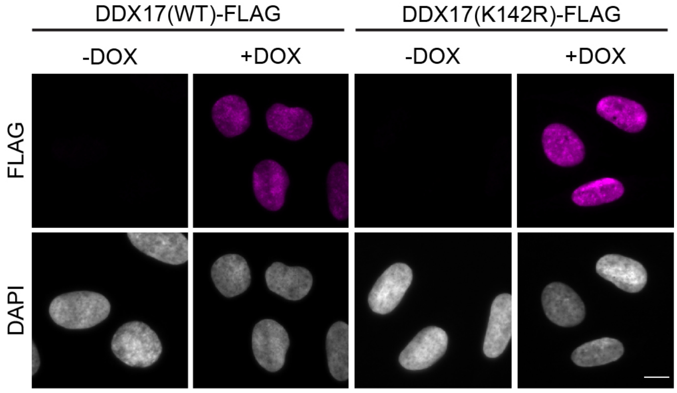

Supplementary Figure S6. Expression of DDX17(WT)-FLAG or DDX17(K142R)-FLAG in U-2 OS T-REx cells.

Cells were treated or not with doxycycline (0.5 ng/ml) for 24 h. Expression of the DDX17 transgenes was assessed by immunofluorescence staining using anti-FLAG antibody. Nuclei were counterstained with DAPI. Scale bar, 10 µm.
